# Numerical analysis for thermal–hydraulic characteristics and the laminar two-phase nanofluid flow inside a tube equipped with helically twisted tapes as swirl and turbulence promoters

**DOI:** 10.1038/s41598-021-91699-x

**Published:** 2021-06-09

**Authors:** Iman Kalani, Davood Toghraie

**Affiliations:** Department of Mechanical Engineering, Khomeinishahr Branch, Islamic Azad University, Khomeinishahr, Iran

**Keywords:** Mechanical engineering, Engineering, Mathematics and computing

## Abstract

In this study, the numerical simulation of heat transfer of Al_2_O_3_-water nanofluid in a pipe equipped with helically twisted tapes is investigated. The volume fraction of nanoparticles in this study is equal to 0, 1, 2, and 3%, and a two-phase mixture method has been used to simulate the nanofluids. The flow regime is laminar in the present study, and Reynolds numbers are *Re* = 250, 500, 750, and 1000. The helical twisted tapes are in three different types, single, double, and triple. The same heat flux 5000Wm^-2^ is applied to the walls. The simulation results showed that increasing the *Re* increases the Nusselt number and decreasing the friction factor. Nusselt number in case 1 and volume fraction of nanoparticles 0% for Re = 250, 500, 750 and 1000 are equal to 95.8, 57.11, 56.13 and 22.15, respectively. The average friction factor is equal to 0.18, 0.09, 0.07, and 0.05. The presence of helical twisted tapes increases the ${\text{Nu}}_{ave}$. The friction factor due to secondary flows and increases the contact of the fluid and the solid surface, so that the Nusselt number in volume fraction of nanoparticles 0%, Re = 250 for case 1, case 2, case 3, and case 4 are 95.8, 46.10, 58.11, and 51.12, respectively, and the friction factor are 18.0, 29.0, 0.38 and 0.48, respectively.

## Introduction

In recent years, attention to the issue of heat transfer improvement in the engineering and industrial sciences has been growing at an increasing rate, so that it has now become a significant part of empirical and theoretical research. Improving heat transfer using conventional methods has resulted in substantial savings in energy costs and resources and environmental protection. Disruption of the laminar sub-layer, creating a secondary flow, reconnecting the separated fluid to the surface delaying the development of the boundary layer, are the most important mechanisms that lead to increased heat transfer through a fluid flow^1^.

Saha et al.^2^ experimentally tested a circular tube with twisted tapes. They concluded that at high *Re*, the twisted elements performed better than the long twisted strips. Chun et al.^3^ examined the effect of Al_2_O_3_ nanoparticles on the heat transfer rate in the heat exchangers with laminar flow experimentally. They found that the $\phi$ and geometric shapes of nanoparticles were the main factors in improving the heat transfer. Date and Saha^4^ examined the flow in a tube equipped with a long twisted strip. They concluded that a significant hydraulic performance could be achieved by reducing the spiral diameter and providing more rotations. Wen and Ding^5^ performed a laboratory study for Al_2_O_3_- water nanofluid in a tube. They studied the thermal efficiency for different *Re*. Sharma et al.^6^ examined a circular tube with a twisted strip. They examined the heat transfer coefficient and the friction factor for the water-Al_2_O_3_ nanofluid. They found that using Al_2_O_3_ nanoparticles in water could significantly increase the heat transfer. Murugesan et al.^7^ examined the friction factor of heat exchangers with twisted strips in a laboratory (water as the operating fluid). They found that the ${\text{Nu}}_{ave}$ and the friction factor for the tube with the square twisted strips were significantly higher than for the simple tube and the tube equipped with the simple twisted bands. Jaisankar et al.^8^ examined the heat transfer characteristics of solar water heaters with twisted strips. They concluded that as the *Re* increased, the turbulence inside the pipe and the heat transfer increased. Salman et al.^9^ conducted numerical research on heat transfer in rotational flow conditions. The results showed that the increase in heat transfer coefficient and friction factor in a pipe with a thick double-sided strip is directly related to the reduction of the torsion coefficient and the cutting depth. Salman et al.^10^ conducted numerical research on nanofluid flow in a circular tube with twisted strips. The results showed that the rate of heat transfer and the friction factor increased with increasing torsion and decreasing the cutting depth of the strips. Sun et al.^11^ used CuO nanoparticles and a helical torsion band to increase heat transfer. They found that in tubes with twisted tapes, the heat transfer coefficient was about twice as large as the horizontal tube heat transfer coefficient and 10 times the flow resistance coefficient. Hong et al.^12^ tested the flow in a simple tube using multiple twisted strips. They concluded that heat transfer could be attributed to rotational flow. Hong et al.^13^ modeled a circular tube with a grooved screw strip. They concluded that the simultaneous use of a grooved screw strip (SGT) and a simple screw strip led to an increase in heat transfer over a simple grooved screw strip. Heat transfer and pressure drop in a heat exchanger equipped with different types of twisted strips was studied in different Refs.^14–16^. Maddah et al.^17^ conducted a study on the water- Al_2_O_3_ nanofluid turbulent flow in horizontal pipes equipped with twisted strips. They found that the ratio of the twisted strip and the concentration of nanoparticles had significant effects on increasing heat transfer and friction factor. Eiamsa-ard and Wongcharee^18^ examined the increase in nanofluid heat transfer in a tube with a twisted strip. They concluded that the heat transfer coefficient and friction factor was directly related to the reduction of the torsion ratio for twisted strips. Jafaryar et al.^19^ examined the increase in nanolfuid heat transfer in a pipe using a rotating strip with an alternating axis. The results showed that the temperature gradient increases with increasing twisting angle, but decreases with pressure. Esfe et al.^20^ studied the increase in heat transfer in a three-edged pipe equipped with twisted strips. They found that the higher the diameter of the three-edged tube, the higher *Re*, the higher the friction factor. Various experimental and numerical studies have been conducted to study the effect of using nanofluid inside pipes, ducts, channels and pipes, mainly in two dimensions and sometimes simplifications in three dimensions. According to the aforementioned reviews, it has been found that nanofluid flow in three-dimensional with two-phase model inside a tube with helical twisted tapes has received less attention from researchers. Also, the number of twisted tapes and the comparison of the results obtained from the two-phase simulation of nanofluid flow are among the items that have received less attention. Therefore, simulation of the number of twisted taps inside the pipe and investigation their performance evaluation criterion is among the current research innovations.

## Numerical method

### Definition and schematic of the problem

**Figure 1:**
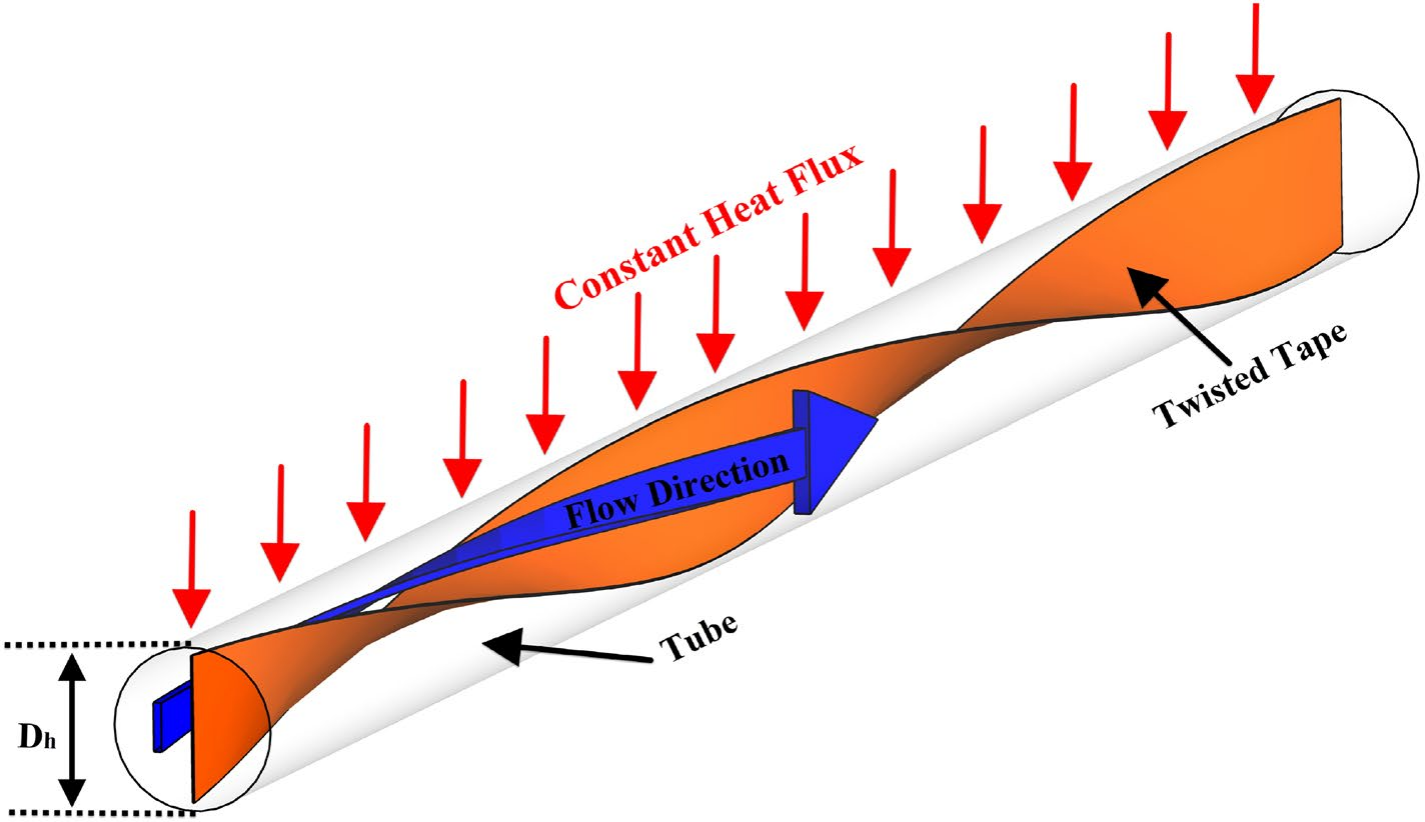
Schematic of the problem.

**Figure 2:**
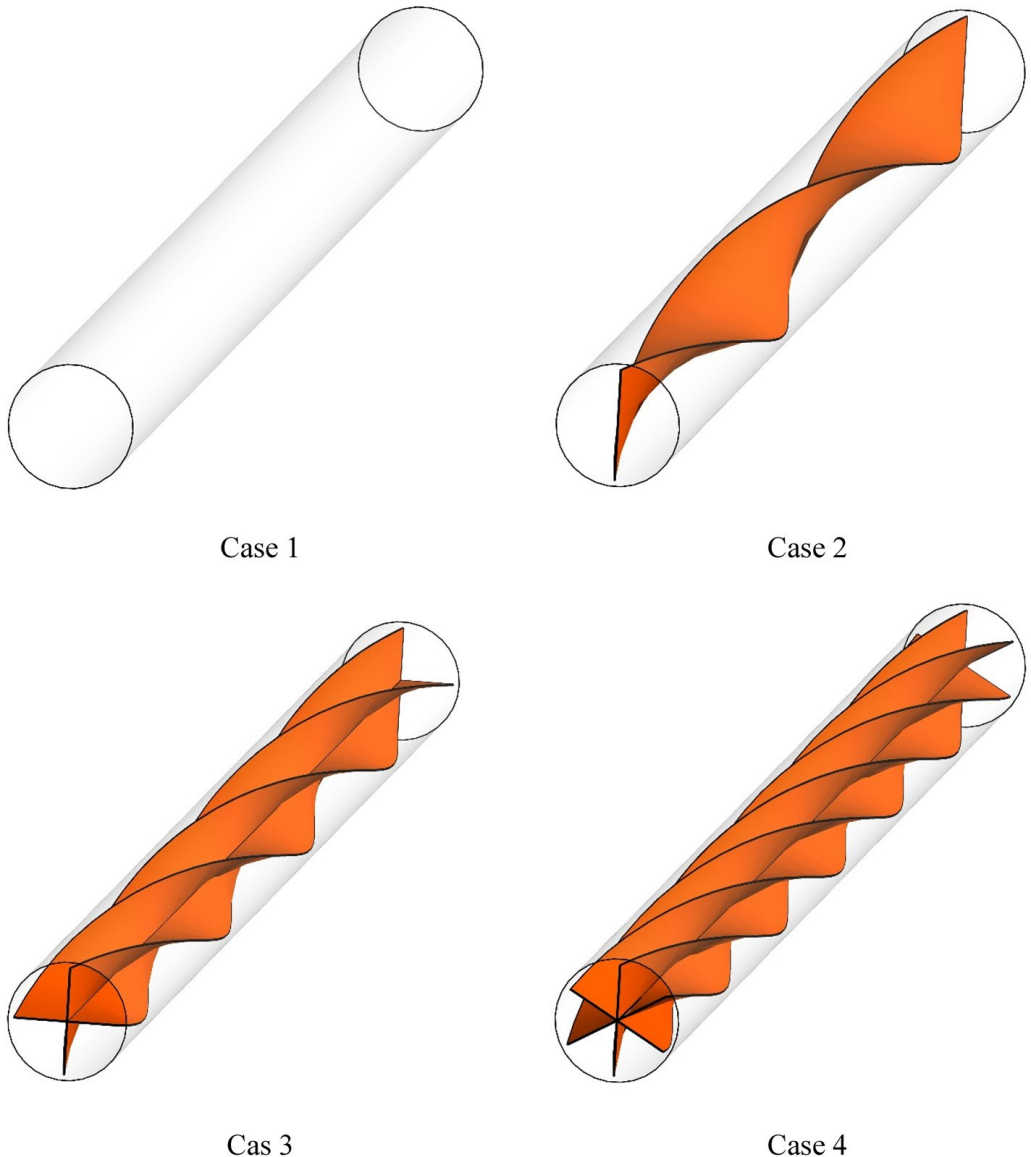
Introducing different geometric cases.

The nanofluid is modeled in a two-phase manner using a mixture method, and the $\phi$ is considered so that the nanofluid remains Newtonian. The volume fraction of nanoparticles studied in this study is equal to $\phi =$ 0, 1, 2, and 3%. The laminar flow regime is simulated in Re = 250, 500, 750, and 1000. The tapes in this study are adiabatic and their height is 80% of the hydraulic diameter of the pipe and their pitch is 400 mm. The diameter of the pipe is 20 mm and the heat flux of 5000 W/m^2^ is applied uniformly to the outer walls of the pipes. The schematic is shown in Fig. 1. Figure 2 shows the different geometric shapes.

Table 1 shows the geometric information such as the number of spiral tapes and the pitch of helical twisted tapes in different cases, which are tabulated to complete the information provided for Figs. 1 and 2.

**Table 1:** The geometric information.

Case	Number of spiral tapes	Pitch [mm]
1	0	–
2	1	400
3	2	400
4	3	400

### Nanofluid properties

In the present study, water- Al_2_O_3_ nanofluid has been used as the operating fluid. The following equations are used to calculate the properties of nanofluid,

Density^19^:

1 $$ \rho_{nf} = \left( {1 - \phi } \right)\rho_{f} + \phi \rho_{s} $$

Specific heat capacity^20^,

2 $$ \left( {\rho C_{p} } \right) = \left( {1 - \phi } \right)\left( {\rho C_{p} } \right)_{f} + \phi \left( {\rho C_{p} } \right)_{s} $$

Dynamic viscosity^21^,

3 $$ \mu_{nf} = \frac{{\mu_{f} }}{{\left( {1 - \phi } \right)^{2.5} }} $$

Thermal conductivity^21^,

4 $$ k_{nf} = 1 + 2.72\phi + 4.97\phi^{2} $$

### Mixture two-phase model

The mixture two-phase model, which is a completely simplified two-phase model, has been used to model the Al_2_O_3_-water nanofluid. The mixture model models two phases (fluid and particles). Also, if the phases are moving at different velocities, a mathematical expression for relative velocity is introduced^22^.

Conservation of mass equation

$$ \overrightarrow {\nabla } \cdot \left( {\rho_{m} \overrightarrow {V}_{m} } \right) = 0 $$

Momentum equation

6 $$ \begin{aligned} \overrightarrow {\nabla } .\left( {\rho_{m} \overrightarrow {V}_{m} \overrightarrow {V}_{m} } \right) & = - \overrightarrow {V} p + \overrightarrow {V} \cdot \left[ {\mu_{m} \left( {\overrightarrow {V} \overrightarrow {V}_{m} + \overrightarrow {V} \overrightarrow {V}_{m}^{T} } \right)} \right] \\ \, & \quad+ \rho_{m} \overrightarrow {g} + \overrightarrow {F} - \overrightarrow {V} \cdot \left( {\sum\limits_{k = 1}^{n} {\phi_{k} \rho_{k} \overrightarrow {V}_{dr,k} \overrightarrow {V}_{dr,k} } } \right) \\ \end{aligned} $$

where $\overrightarrow {F}$ is volumetric forces, $\mu_{m}$ is the effective viscosity of the mixture, $n$ is the number of phases and $\overrightarrow {V}_{dr,k}$ is the driving velocity of the secondary phase.

7 $$ \overrightarrow {V}_{dr,k} = \overrightarrow {V}_{k} - \overrightarrow {V}_{m} $$

Energy equation

8 $$ \overrightarrow {\nabla } \cdot \left[ {\sum\limits_{k = 1}^{n} {(\rho_{k} c_{pk} )\phi_{k} \mathop {V_{k} }\limits^{ \to } T} } \right] = \overrightarrow {\nabla } \cdot k_{m} \overrightarrow {\nabla } T $$

where $\overrightarrow {V}_{m}$ is the average mass velocity and $\rho_{m}$ is the density of the mixture,

9 $$ \overrightarrow {V}_{m} = \frac{{\sum\limits_{k = 1}^{n} {\phi_{k} \rho_{k} \overrightarrow {V}_{k} } }}{{\rho_{m} }} $$

10 $$ \rho_{m} = \sum\limits_{k = 1}^{n} {\phi_{k} \rho_{k} } $$

In the above relation, $\phi_{k}$ is the volume fraction of k- phase. Also,

11 $$ \mathop {V_{pf} }\limits^{ \to } = \mathop {V_{p} }\limits^{ \to } - \mathop {V_{f} }\limits^{ \to } $$

12 $$ \mathop V\limits^{ \to }_{dr,p} = \mathop {V_{pf} }\limits^{ \to } - \sum\limits_{k = 1}^{n} {\frac{{\phi_{k} \rho_{k} }}{{\rho_{m} }}\mathop {V_{fk} }\limits^{ \to } } $$

The viscosity of the mixture is calculated as follows,

13 $$ \mu_{m} = \sum\limits_{k = 1}^{n} {\phi_{k} \mu_{k} } $$

In the present study, the relative velocity provided by Manin^17^ and the Schiller-Newman drag function^18^ have been used.

14 $$ \mathop V\limits^{ \to }_{pf} = \frac{{\rho_{p} d_{p}^{2} (\rho_{p} - \rho_{m} )}}{{18\mu_{f} f_{drag} \rho_{p} }}\left( {\vec{g} - \left( {\vec{V}_{m} \cdot \nabla } \right)\vec{V}_{m} } \right) $$

15 $$ f_{drag} = \left\{ {\begin{array}{*{20}c} {1 + 0.15{\text{Re}}_{p}^{0.687} } & {\left( {{\text{Re}}_{p} \le 1000} \right)} \\ {0.0183{\text{Re}}_{p} } & {({\text{Re}}_{p} > 1000)} \\ \end{array} } \right. $$

Therefore, the drift velocity is obtained as follows:

16 $$ \overrightarrow {V}_{dr,p} = \overrightarrow {{V_{pf} }} - \sum\limits_{k = 1}^{n} {\left( {\frac{{\phi_{k} \rho_{k} }}{{\rho_{m} }}\overrightarrow {V}_{fk} } \right)} $$

### Definition of Nusselt number and friction factor

17 $$ {\text{Nu}}_{x} = \frac{{hD_{h} }}{{k_{nf} }} $$

18 $$ {\text{Nu}}_{ave} = \frac{1}{L}\int_{0}^{L} {{\text{Nu}}_{{\text{x}}} } dx $$

19 $$ {\text{f}}_{loc} = \frac{2\tau }{{\rho_{nf} U^{2} }} $$

20 $$ {\text{f}}_{ave} = \frac{1}{L}\int_{0}^{L} {{\text{f}}_{loc} } dx $$

where,

21 $$ {\text{D}}_{{\text{h}}} = \frac{4A}{P} $$

Also, in this study, the dimensionless length has been used, which indicates the ratio of the fluid position in the direction of flow to the total length of the pipe and is defined as follows.

22 $$ X = \frac{x}{L} $$

### Assumptions

- The flow is laminar, incompressible, three-dimensional, and steady.
- The properties of nanofluid are a function of $\phi$.
- There is no radiation in the problem and the viscous dissipation is avoided.
- The pipe is made of Aluminum and water is the base fluid.

### Boundary conditions

- *Inlet conditions* Fixed velocities are calculated based on *Re* and properties of nanofluid, and the inlet temperature is also constant.
- *Outlet conditions* The outlet pressure is equal to atmospheric pressure.
- *Conditions of the walls* On all the walls, the no-slip boundary condition is applied. Fixed heat flux 5000Wm^-2^ is applied to the walls.
- *Twisted tapes* On all walls, the no-slip boundary condition is applied and they are also thermally insulated.

### Mesh generation

**Figure 3:**
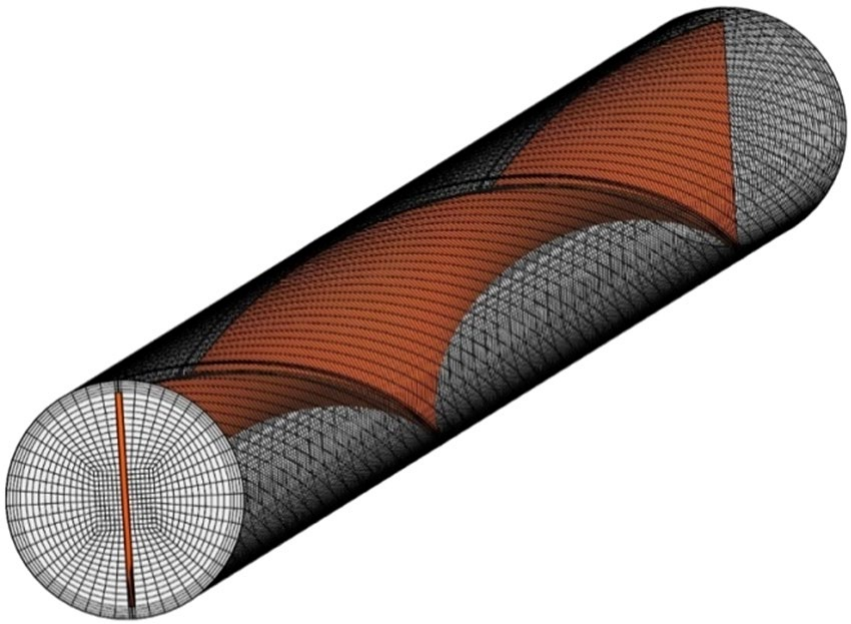
a structured grid for present geometry.

In this research, a structured grid has been used, which is shown in Fig. 3.

### Grid independency

Given that in numerical simulation, the number of computational elements has a significant effect on the obtained results, it is necessary to examine the independency of the solution from the number of computational grids. For this purpose, the ${\text{Nu}}_{ave}$ is shown in Table 2 according to the number of different elements in the flow with the Re = 1000. As can be seen, with the increase in the number of elements to more than 1,350,000 elements, the results obtained have not changed. Then, the 1,350,000 elements have been used in all simulations.

**Table 2:** Specifications of different models for grid independency.

Nmber of computational elements	${\text{Nu}}_{ave}$	Error [%]
250,000	50.67	
600,000	35.01	44.73
1,000,000	26.3	33.11
1,350,000	25.35	3.70
1,750,000	25.21	0.59
2,250,000	25.2	0.03

### Validation

**Figure 4:**
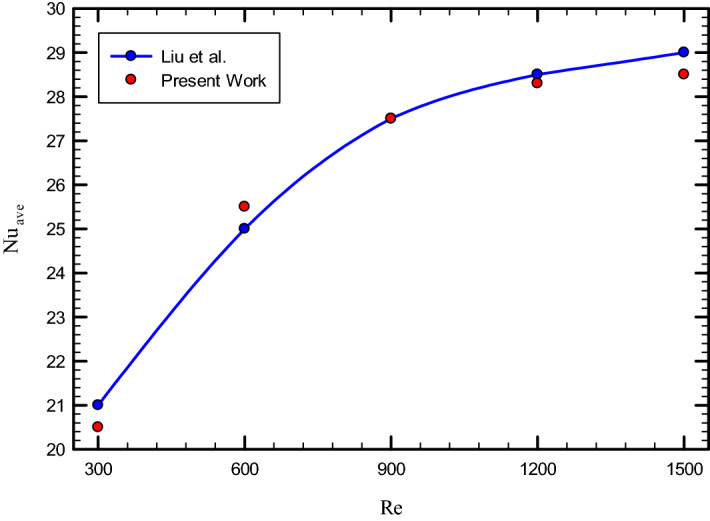
Validation of present work with Ref.^22^.

To ensure the accuracy of results, we compared the obtained results with the valid laboratory results. For this purpose, Ref.^22^ has been used as a validation reference. In this reference, the heat transfer characteristics of pure water flow inside the pipe, despite a twisted strip, are examined numerically and experimentally. Figure 4 compares the obtained ${\text{Nu}}_{ave}$ with the ${\text{Nu}}_{ave}$ provided in Ref.^22^. As can be seen, the obtained results are well compatible with the experimental results (maximum 1.17% error), which shows that the process governing the numerical solution of the problem is very accurate.

### Results and discussion

### The effect of *Re* on ${\text{Nu}}_{x}$

**Figure 5:**
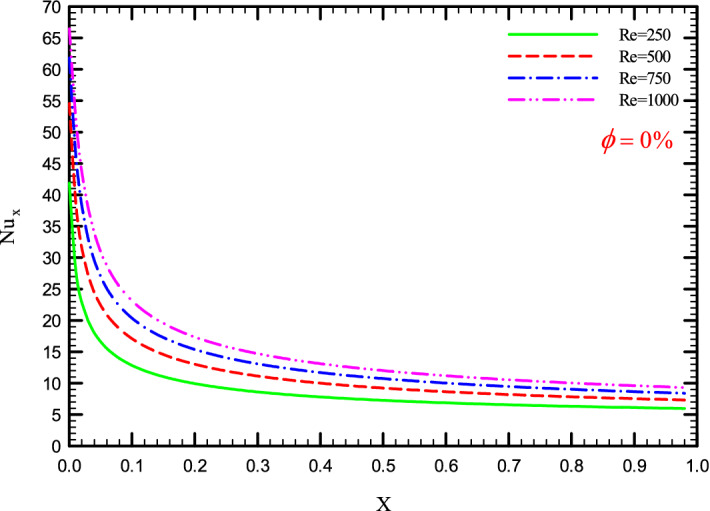
${\text{Nu}}_{x}$ versus dimensionless length in $\phi = 0\%$ for Case 1.

**Figure 6:**
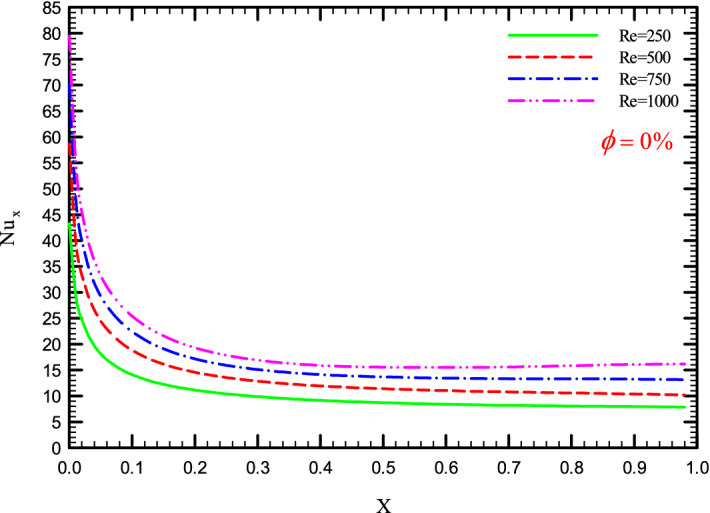
${\text{Nu}}_{x}$ versus dimensionless length in $\phi = 0\%$ for Case 2.

**Figure 7:**
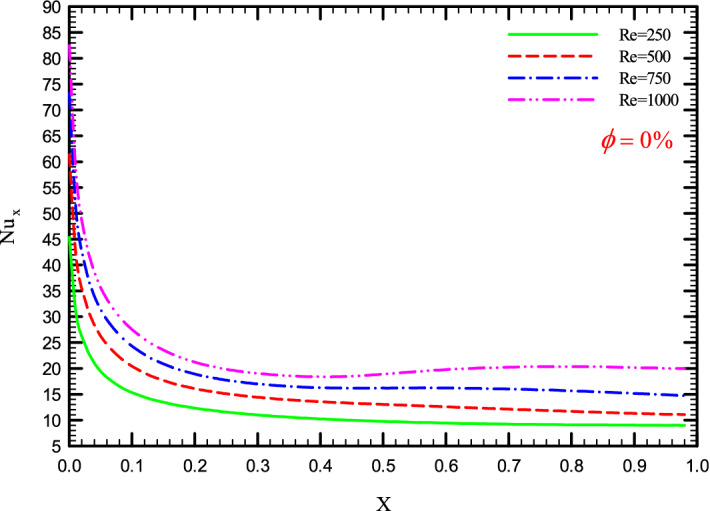
${\text{Nu}}_{x}$ versus dimensionless length in $\phi = 0\%$ for Case 3.

**Figure 8:**
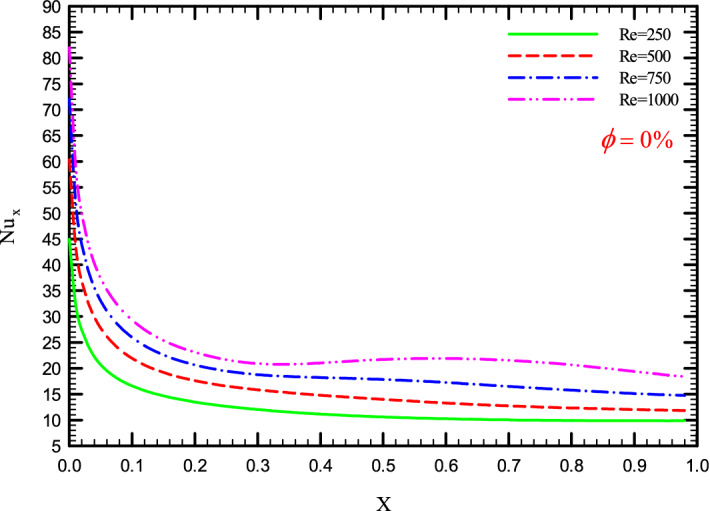
${\text{Nu}}_{x}$ versus dimensionless length in $\phi = 0\%$ for Case 4.

Figures 5, 6, 7 and 8 show a diagram of the local Nusselt number (${\text{Nu}}_{x}$) versus dimensionless length of the tube at Re = 250, 500, 750, and 1000 for different geometric cases. The clear characteristic of the diagram in all cases is that at the beginning of the flow path or at the inlet of the pipe, the ${\text{Nu}}_{x}$ starts from a maximum value and decreases sharply by passing along the flow path and then remains constant. As long as the ${\text{Nu}}_{x}$ is decreasing, the flow is in the developing part, and as the ${\text{Nu}}_{x}$ is fixed, the fluid is placed in the developed zone.

### The effect of Re on ${\text{Nu}}_{ave}$

**Figure 9:**
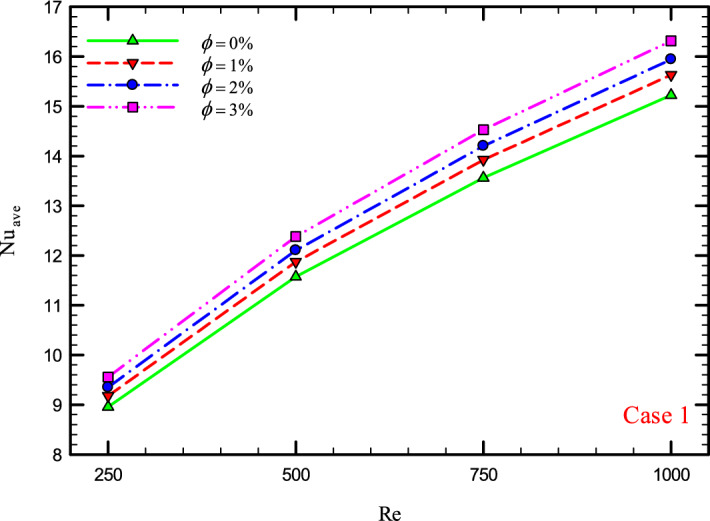
${\text{Nu}}_{ave}$ versus **Re** for Case 1.

**Figure 10:**
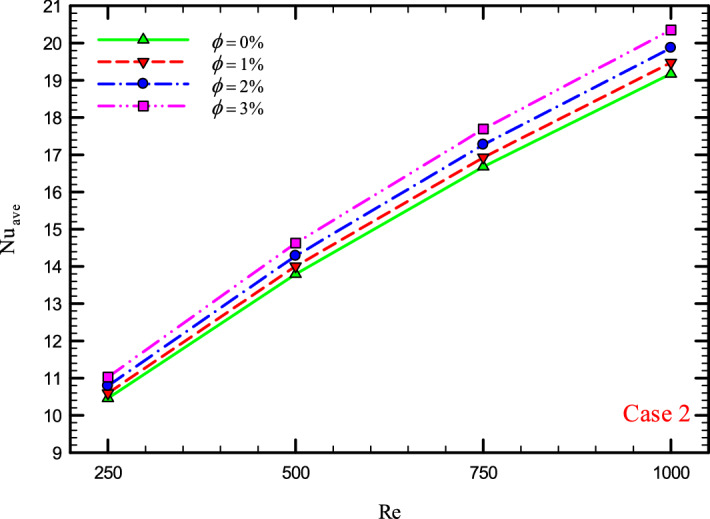
${\text{Nu}}_{ave}$ versus **Re** for Case 2.

**Figure 11:**
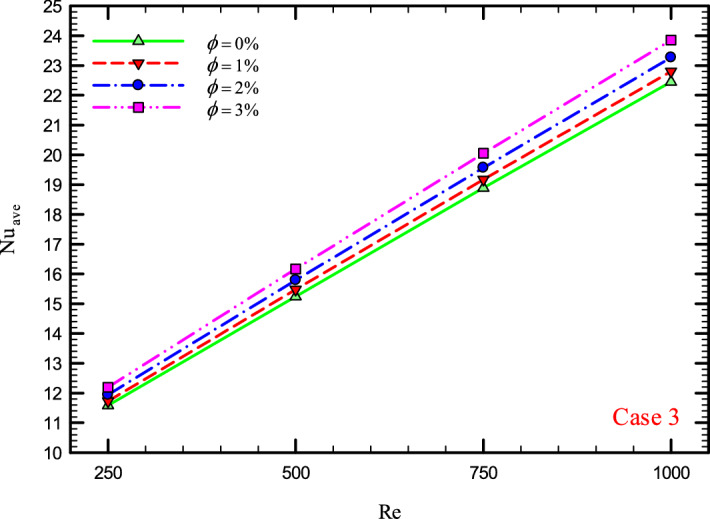
${\text{Nu}}_{ave}$ versus **Re** for Case 3.

**Figure 12:**
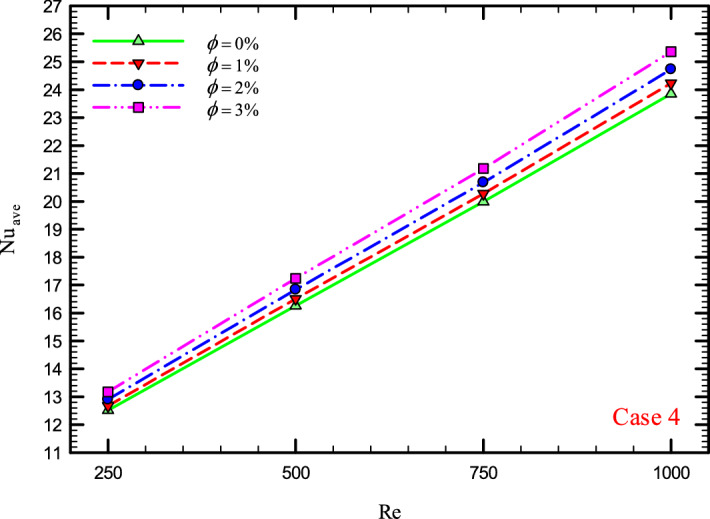
${\text{Nu}}_{ave}$ versus Re for Case 4.

Figures 9, 10, 11 and 12 show the average Nusselt number (${\text{Nu}}_{ave}$) for Re = 250, 500, 750, and 1000 in $\phi =$ 0, 1, 2, and 3% for different geometric cases. As mentioned, increasing the *Re* leads to an increase in the overall level of ${\text{Nu}}_{x}$, and ultimately increases the heat transfer. According to figures, it can be seen that with the increase in the Re, the amount of ${\text{Nu}}_{x}$ increases, and also the time it takes for the flow to reach the developed state is delayed. The reason for the increase in ${\text{Nu}}_{ave}$ with the increase in the Re can be described as the fact that with the increase in the Re, the inertial forces overcome the viscous forces, and as a result the shear stress between the walls and the fluid decreases.

### The effect of Re on surface temperature

**Figure 13:**
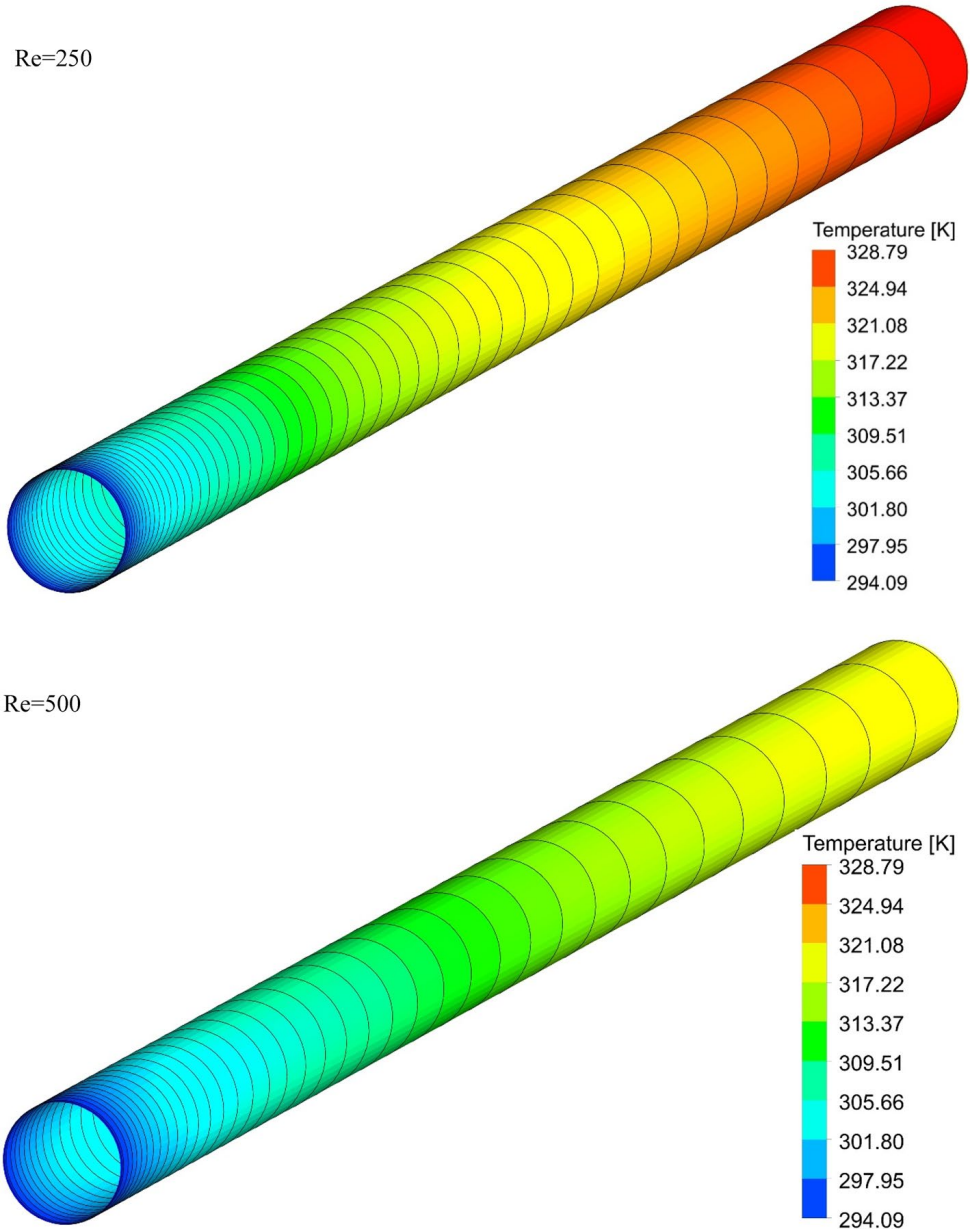

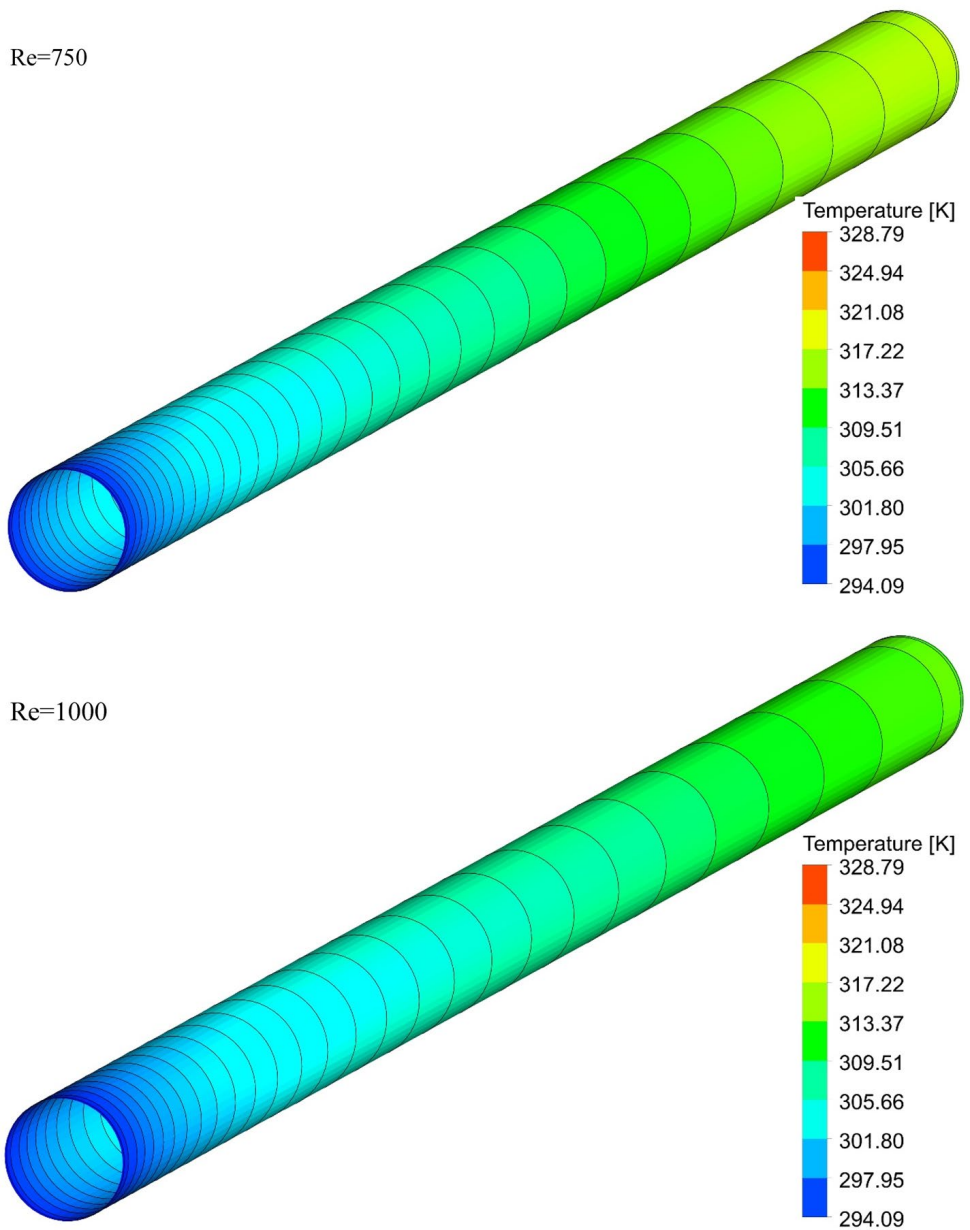
Isotherm contours on the surface of the pipe in $\phi = 0\%$ and Case 1.

**Figure 14:**
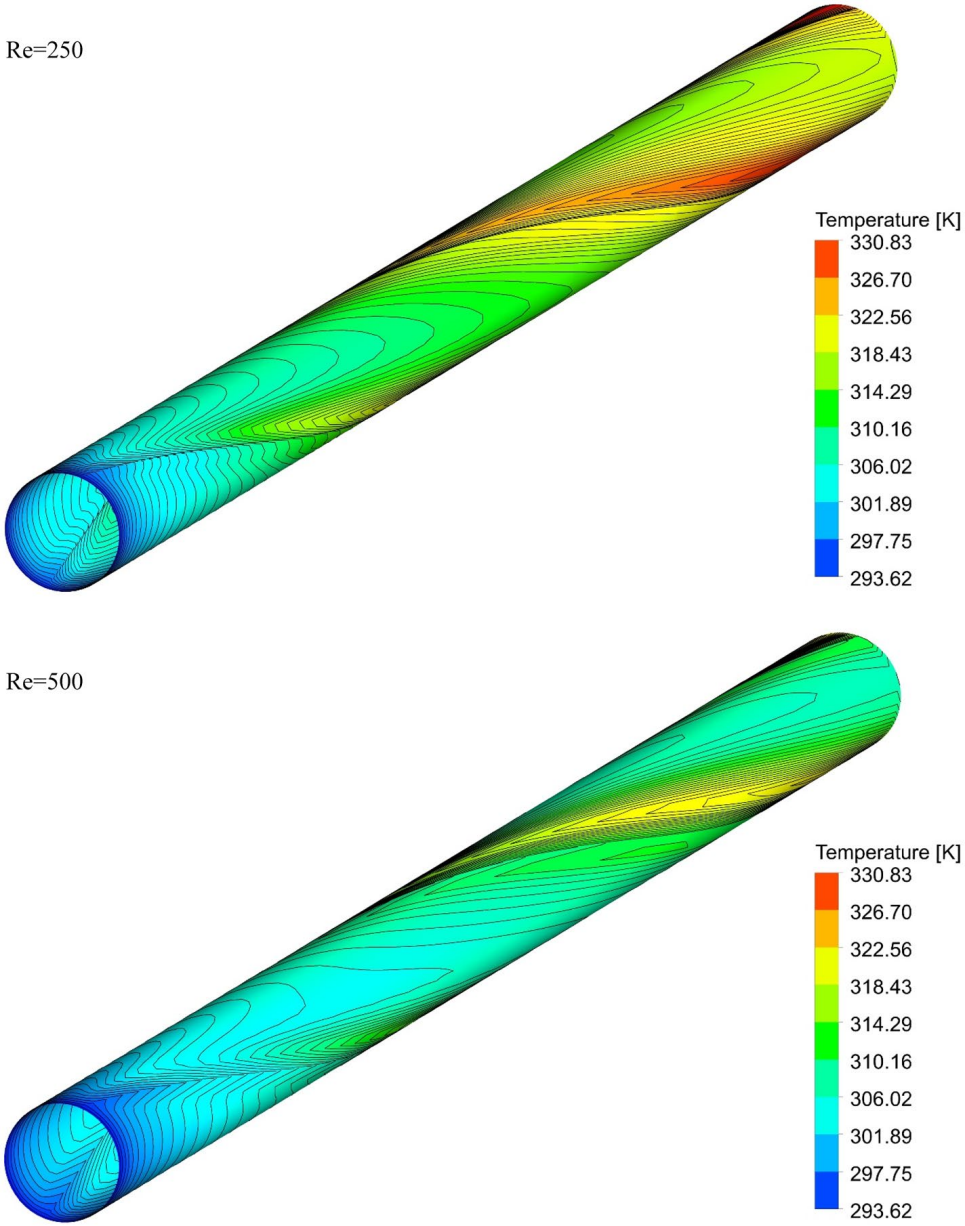

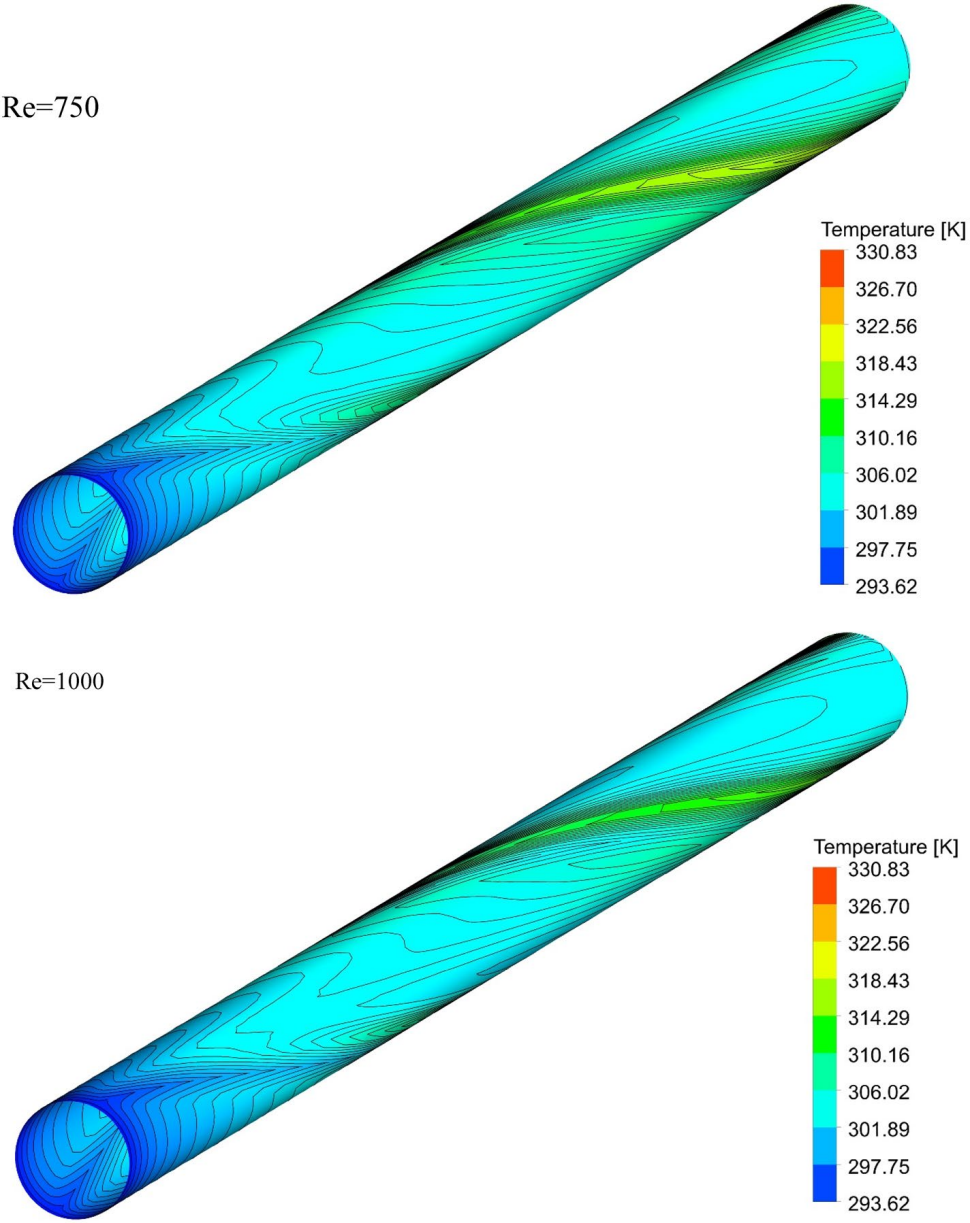
Isotherm contours on the surface of the pipe in $\phi = 0\%$ and Case 2.

**Figure 15:**
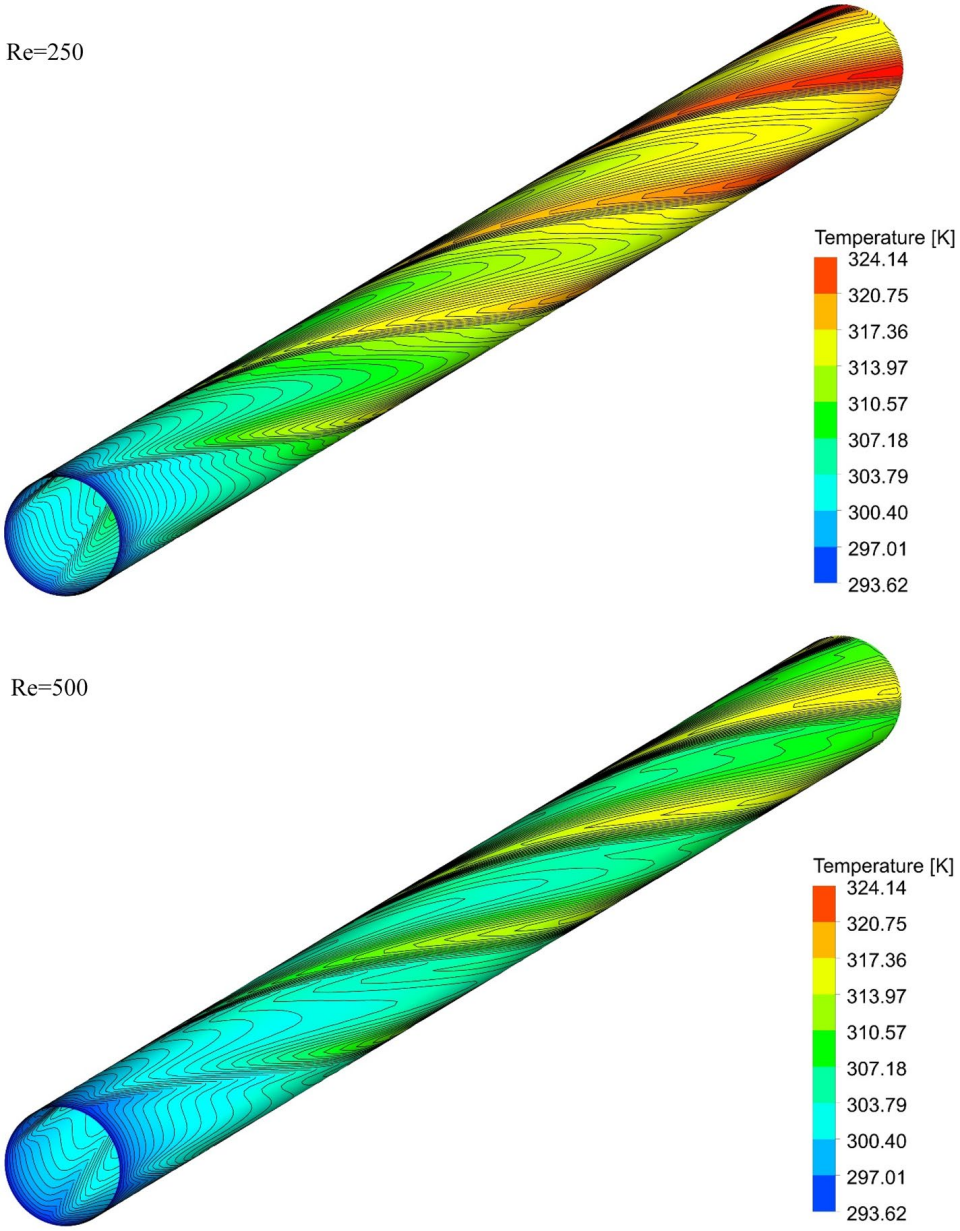

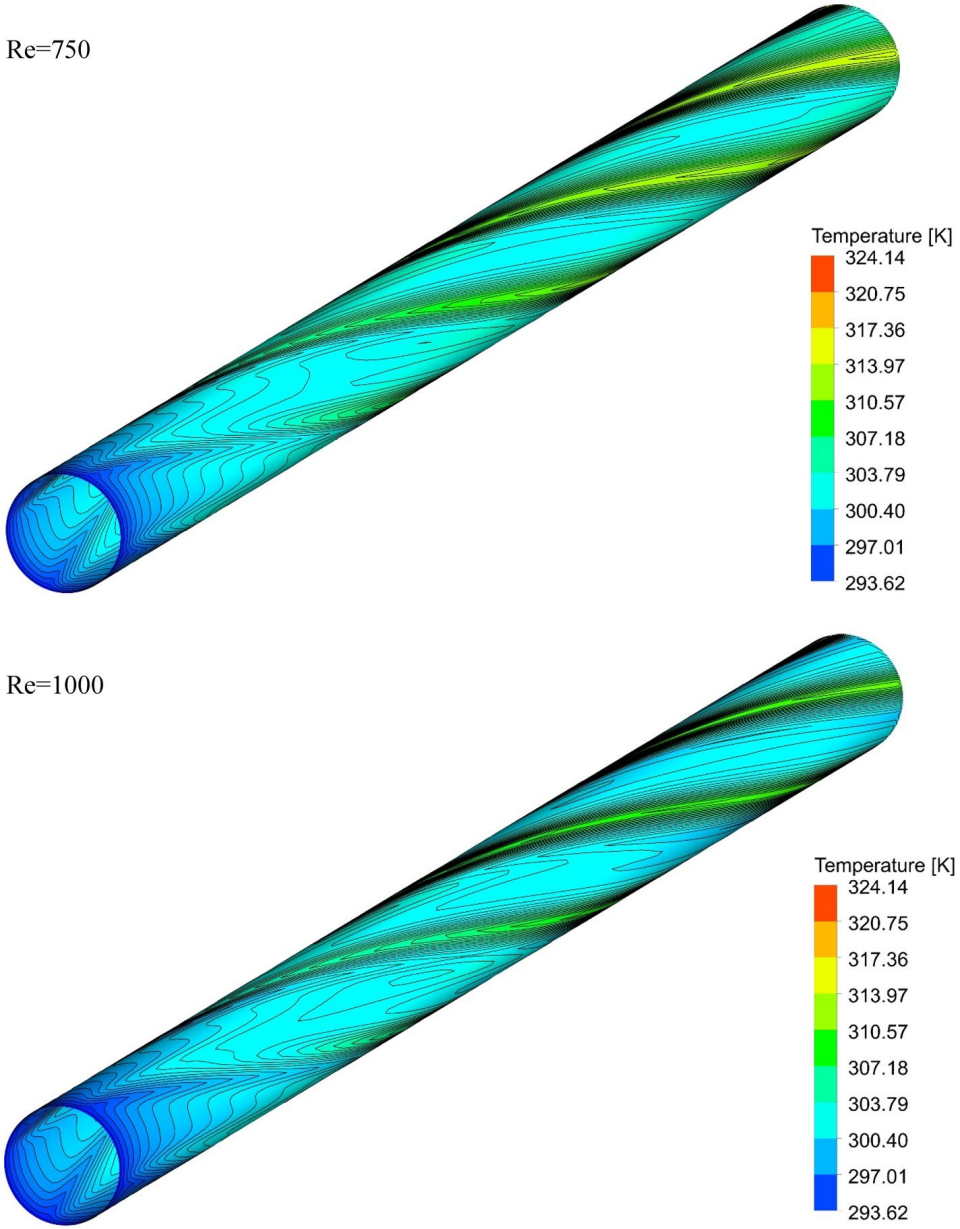
Isotherm contours on the surface of the pipe in $\phi = 0\%$ and Case 3.

**Figure 16:**
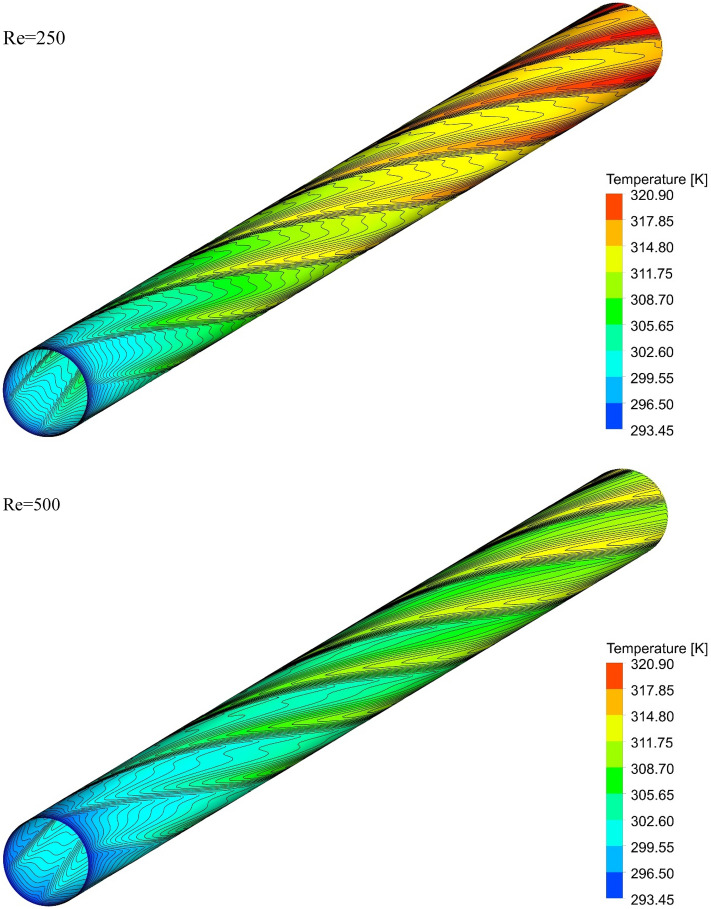

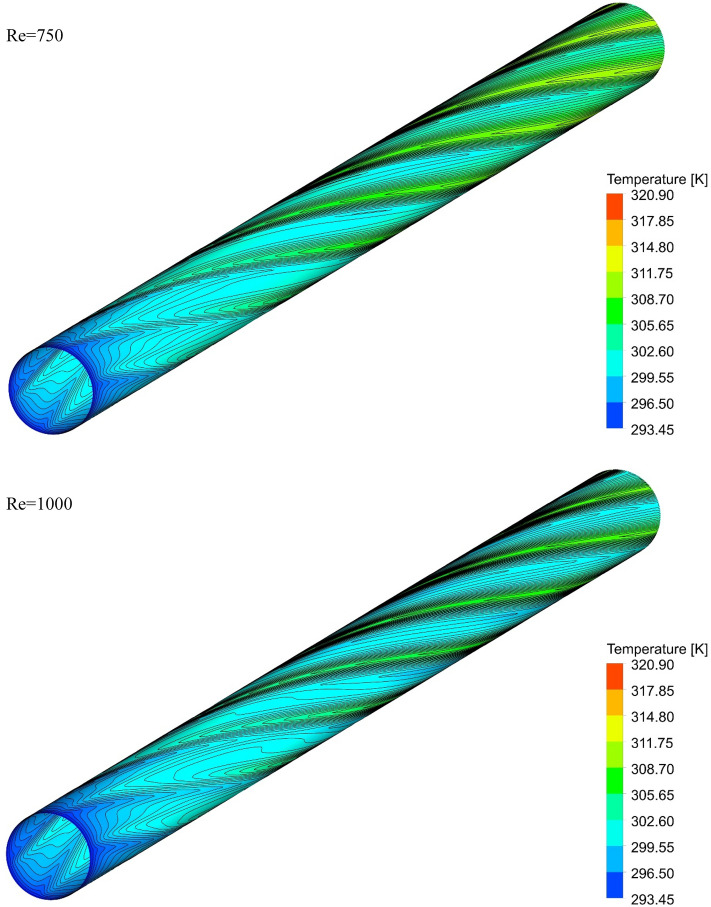
Isotherm contours on the surface of the pipe in $\phi = 0\%$ and Case 4.

Figures 13, 14, 15 and 16 show the temperature contours for the different volume fraction of nanoparticles, Re = 250, 500, 750, and 1000 for different cases. As can be seen, in all cases, the contour density in the inlet area of the pipe is higher than the other points of the pipe, and the distance between the lines increases with the passage along the flow path. It can also be said that the pattern of temperature contours in case 1 is different from other cases that are equipped with helical twisted tapes. It is easy to see that in all geometric cases, as the *Re* increases, the overall temperature of the walls under the heat flux decreases, due to the increase in the heat transfer due to the increase in the *Re*. This improvement is due to the increase in fluid velocity due to the increase in *Re*, which causes the fluid to communicate with the walls in less time, and as a result, the fluid penetrates along the flow path more before it loses its cooling power. Further penetration of cooler fluid along the flow path causes a greater difference in fluid temperature with the walls, which further increases heat transfer or better cooling of the walls.

### Effect of $\phi$ on ${\text{Nu}}_{x}$

**Figure 17:**
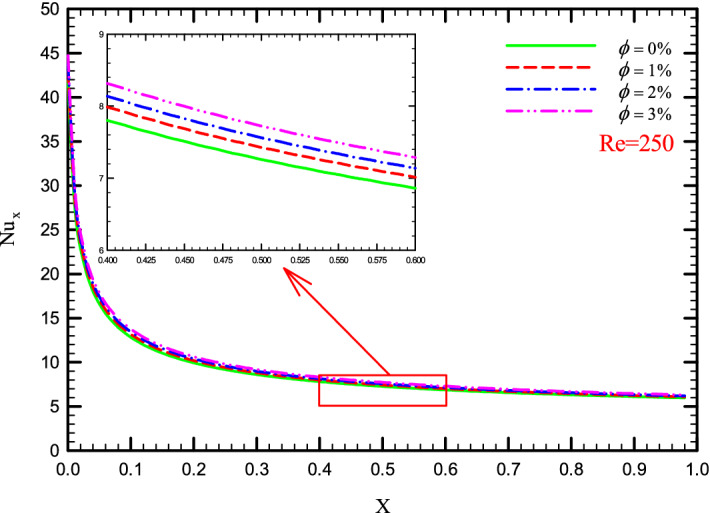
${\text{Nu}}_{x}$ versus dimensionless length in Re = 250 for Case 1.

**Figure 18:**
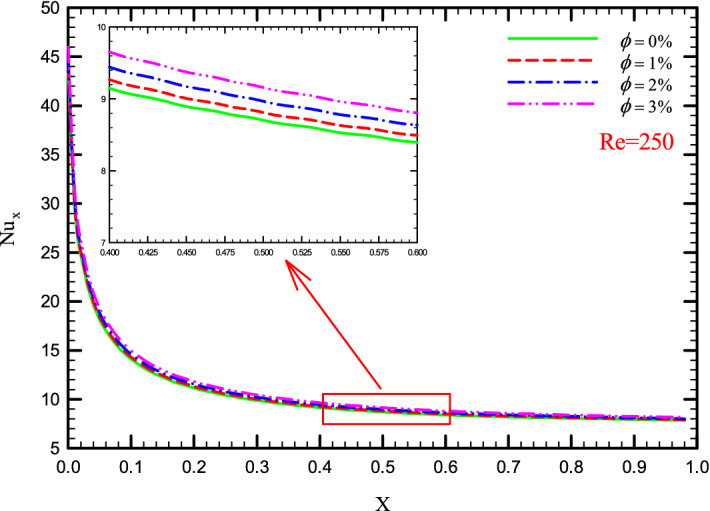
${\text{Nu}}_{x}$ versus dimensionless length in Re = 250 for Case 2.

**Figure 19:**
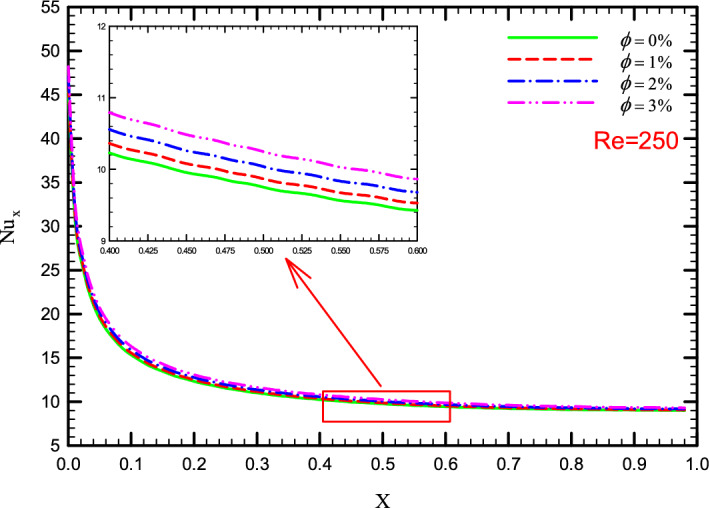
${\text{Nu}}_{x}$ versus dimensionless length in Re = 250 for Case 3.

**Figure 20:**
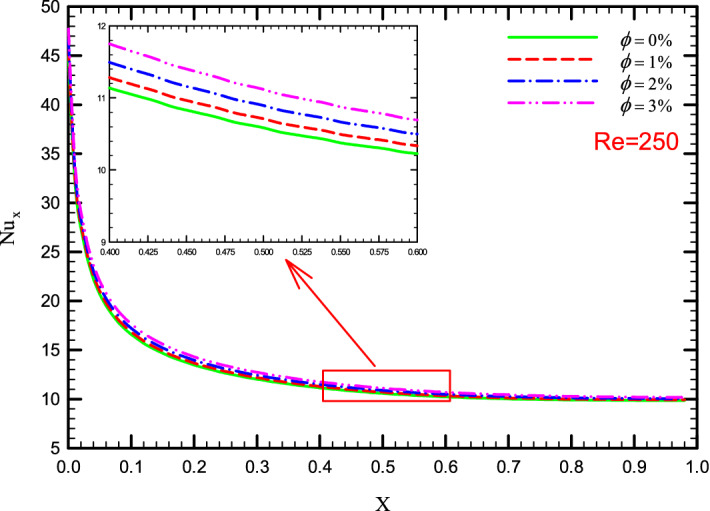
${\text{Nu}}_{x}$ versus dimensionless length in Re = 250 for Case 4.

Figures 17, 18, 19 and 20 show the ${\text{Nu}}_{x}$ versus dimensionless length for Re = 250, $\phi =$ 0, 1, 2, and 3% for different geometric cases. As with the increase in *Re*, which increases the overall level of the ${\text{Nu}}_{x}$, it can be seen that increasing the $\phi$ also leads to an increase in the general level of the ${\text{Nu}}_{x}$ chart, except that the difference is due to the increase in the $\phi$ is smaller compared to the difference created by the increase in the *Re*. The increase in the ${\text{Nu}}_{x}$ due to the addition of nanoparticles to the base fluid can be attributed to the improvement of the properties of the base fluid.

### The effect of $\phi$ on ${\text{Nu}}_{ave}$

**Figure 21:**
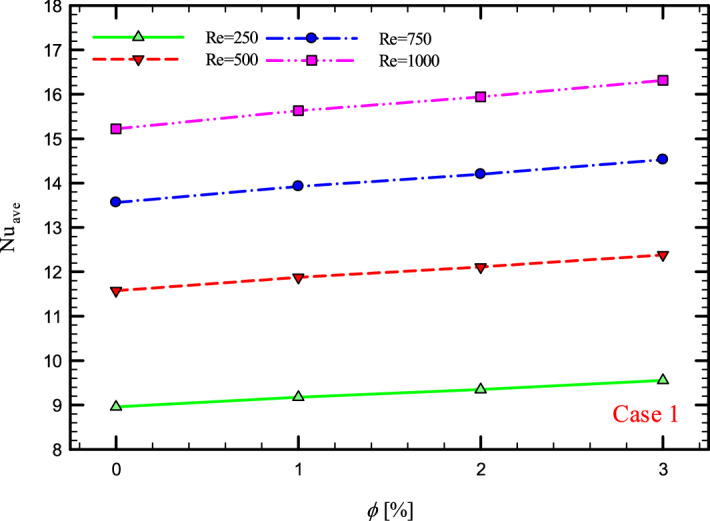
${\text{Nu}}_{ave}$ versus $\phi$ for Case 1.

**Figure 22:**
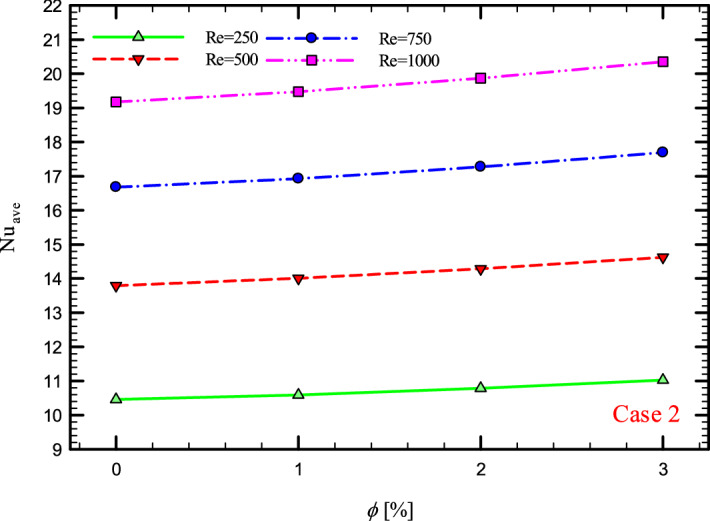
${\text{Nu}}_{ave}$ versus $\phi$ for Case 2.

**Figure 23:**
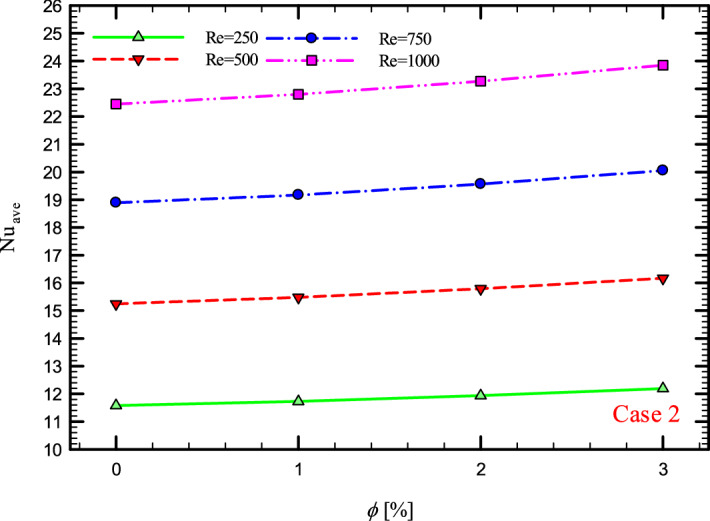
${\text{Nu}}_{ave}$ versus $\phi$ for Case 3.

**Figure 24:**
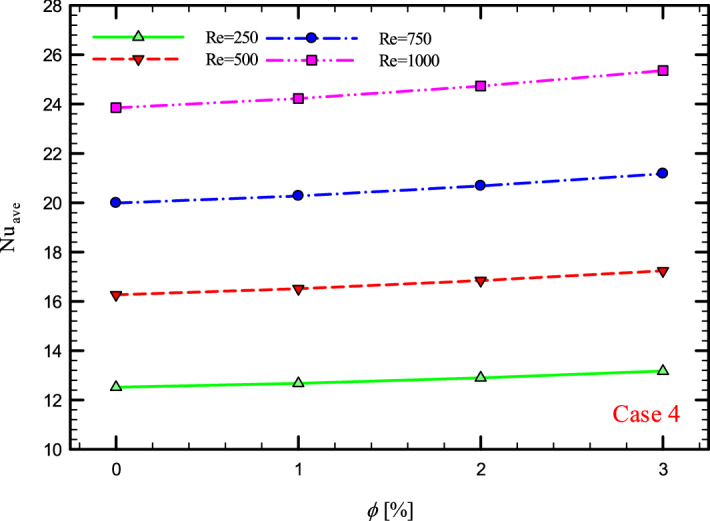
${\text{Nu}}_{ave}$ versus $\phi$ for Case 4.

Figures 21, 22, 23 and 24 show the graph of the $${\text{Nu}}_{ave}$$ versus $$\phi$$ in Re = 250, 500, 750 and 1000, $$\phi =$$ 0, 1, 2 and 3% and for different cases. The highest rate of $${\text{Nu}}_{ave}$$ in each *Re* and all geometric cases is related to the highest $$\phi$$$$\left( {\phi = 3\% } \right)$$ and the lowest amount related to pure water or $$\phi =$$ 0%. Increasing the thermal conductivity of nanofluids is one of the main factors in this increase in heat transfer. In addition to increasing the thermal conductivity, reducing thermal resistance is also one of the factors that improve the properties of the fluid. Because by reducing the thermal resistance of the operating fluid, the heat from the walls penetrates more easily into the depth of the fluid, and as a result, the nanofluid gains more heat from the wall in less time, which leads to better cooling and increased heat transfer.

### Effect of the number of twisted tapes on ${\text{Nu}}_{loc}$

**Figure 25:**
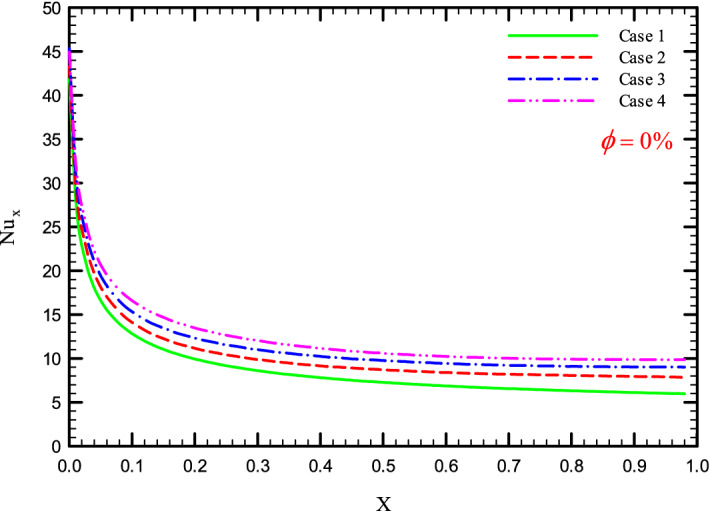
${\text{Nu}}_{loc}$ versus dimensionless length in $\phi = 0\%$ in Re = 250.

**Figure 26:**
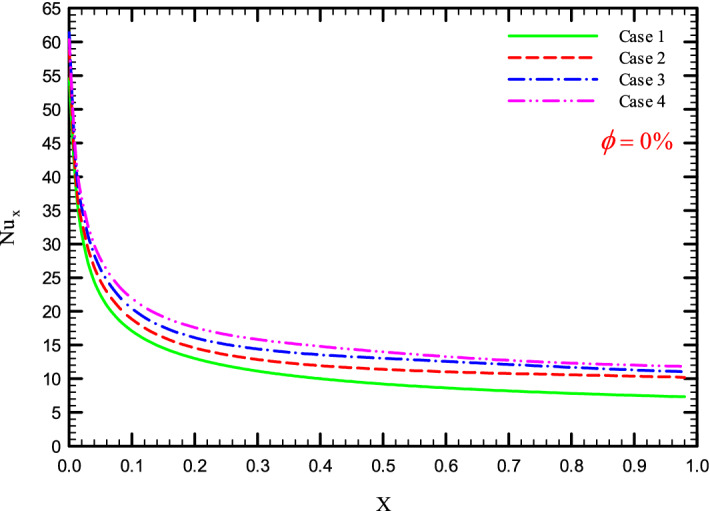
${\text{Nu}}_{loc}$ versus dimensionless length in $\phi = 0\%$ in Re = 500.

**Figure 27:**
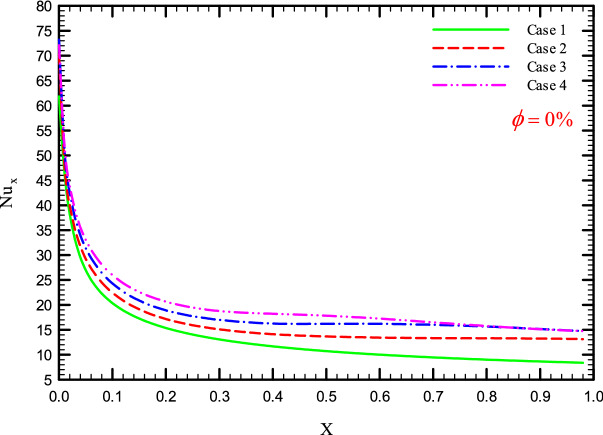
${\text{Nu}}_{loc}$ versus dimensionless length in $\phi = 0\%$ in Re = 750.

**Figure 28:**
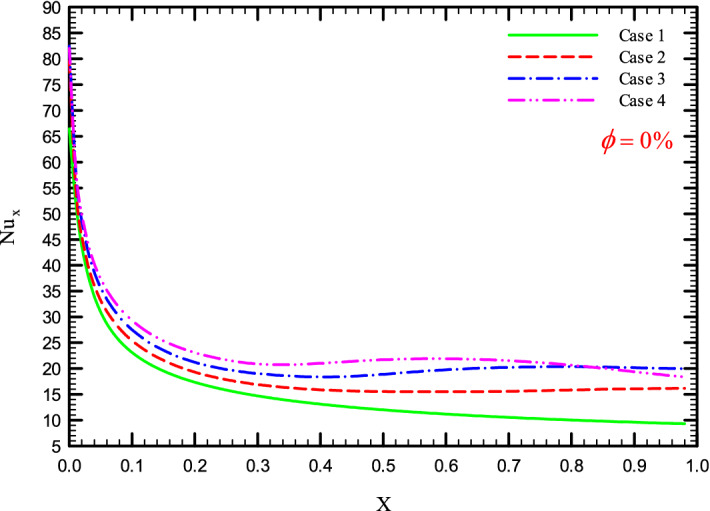
${\text{Nu}}_{loc}$ versus dimensionless length in $\phi = 0\%$ in Re = 1000.

Figures 25, 26, 27 and 28 show the ${\text{Nu}}_{loc}$ versus dimensionless length for Re = 250, 500, 750, and 1000, and different $\phi$ and geometric cases. It can be seen that the presence of the helical twisted tapes increases the overall level of the ${\text{Nu}}_{loc}$, and this is exacerbated by the increase in the number of twisted tapes. Among the factors that can be mentioned for this increase in heat transfer is that: the presence of the twisted tapes causes the surface of the tube to shrink, and as a result, in a fixed *Re*, the fluid velocity increases to satisfy the conservation of mass. As a result, surface cooling and heat transfer increase. One of the main reasons for the increase in heat transfer in pipes equipped with twisted tapes is the presence of secondary flows caused by the twisted tapes, which create better mixing. The presence of these secondary flows causes the viscous sub-layers to be disrupted, thereby reducing the thickness of the boundary layer, destroying the boundary layer, and delaying the formation of boundary layers.

### The effect of the number of helical twisted tapes on ${\text{Nu}}_{ave}$

**Figure 29:**
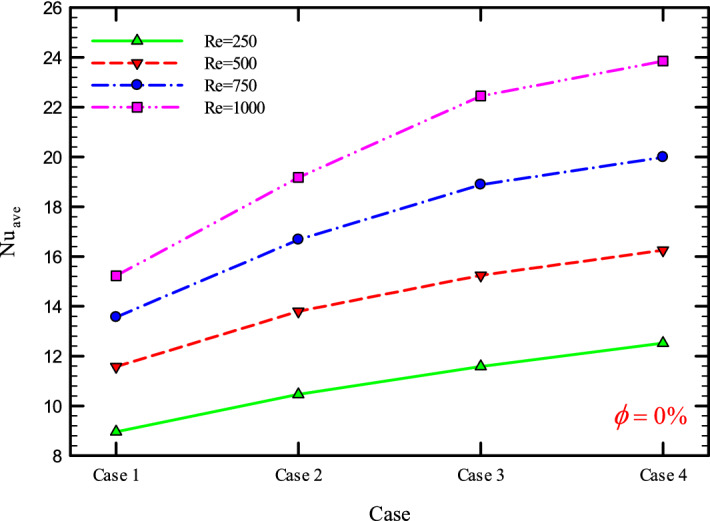
${\text{Nu}}_{ave}$ for different cases for $\phi = 0\%$.

**Figure 30:**
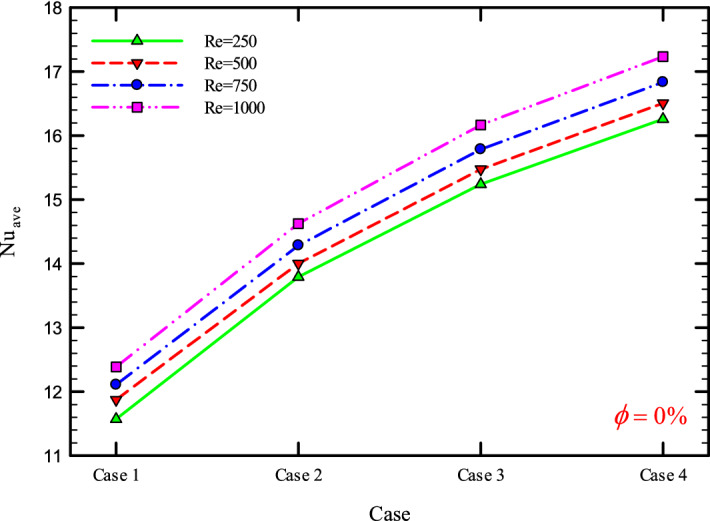
${\text{Nu}}_{ave}$ for different cases for $\phi = 1\%.$

**Figure 31:**
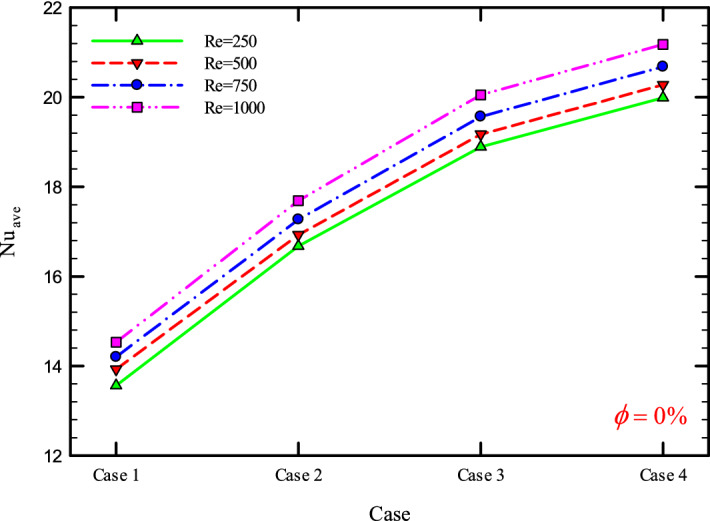
${\text{Nu}}_{ave}$ for different cases for $\phi = 2\%.$

**Figure 32:**
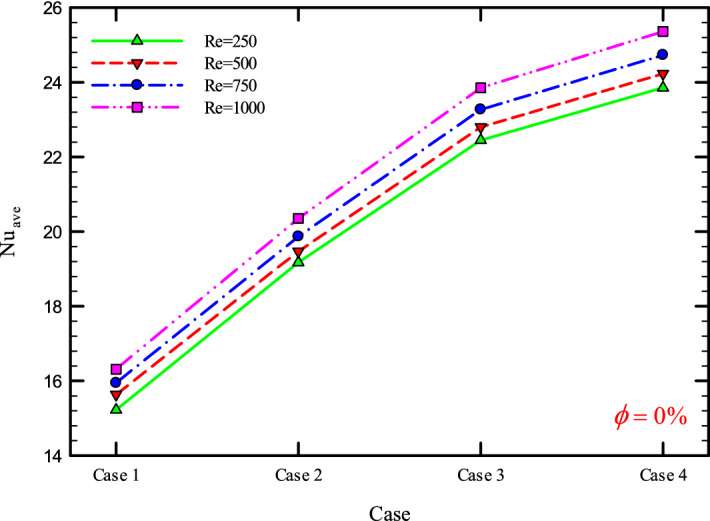
${\text{Nu}}_{ave}$ for different cases for $\phi = 3\%.$

**Figure 33:**
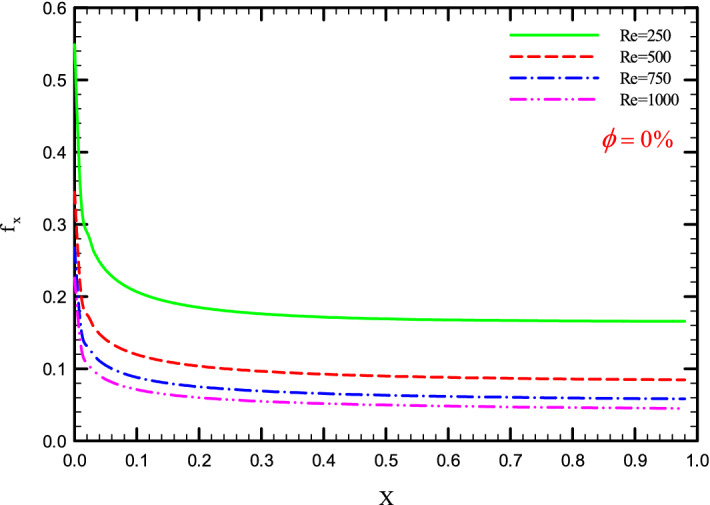
$f_{x}^{{}}$ versus dimensionless length in $\phi = 0\%$ for Case.

**Figure 34:**
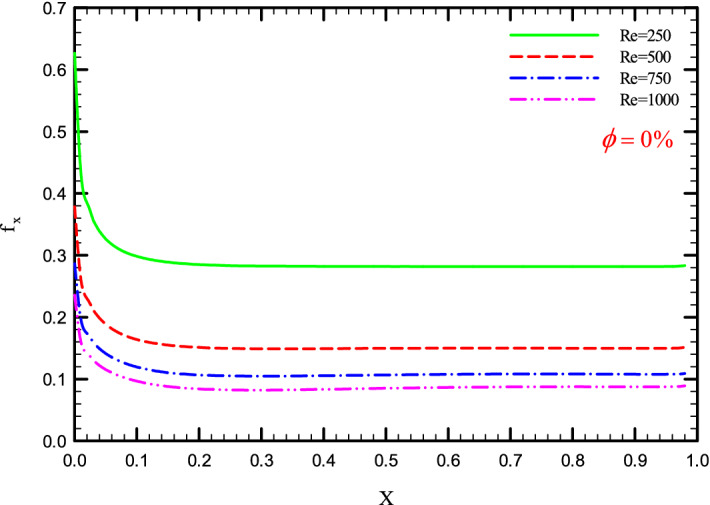
$f_{x}^{{}}$ versus dimensionless length in $\phi = 0\%$ for Case 2.

**Figure 35:**
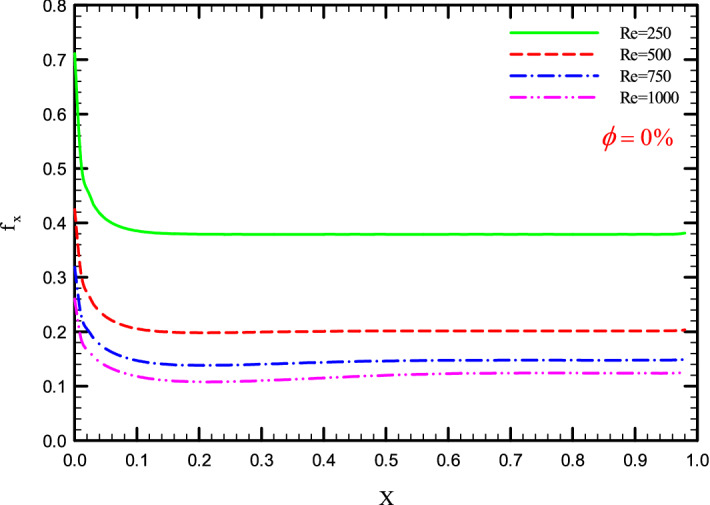
$f_{x}^{{}}$ versus dimensionless length in $\phi = 0\%$ for Case 3.

**Figure 36:**
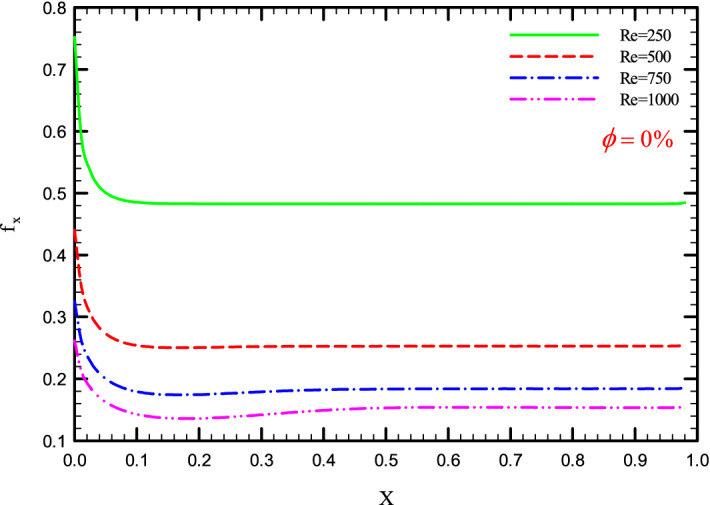
$f_{x}^{{}}$ versus dimensionless length in $\phi = 0\%$ for Case 4.

**Figure 37:**
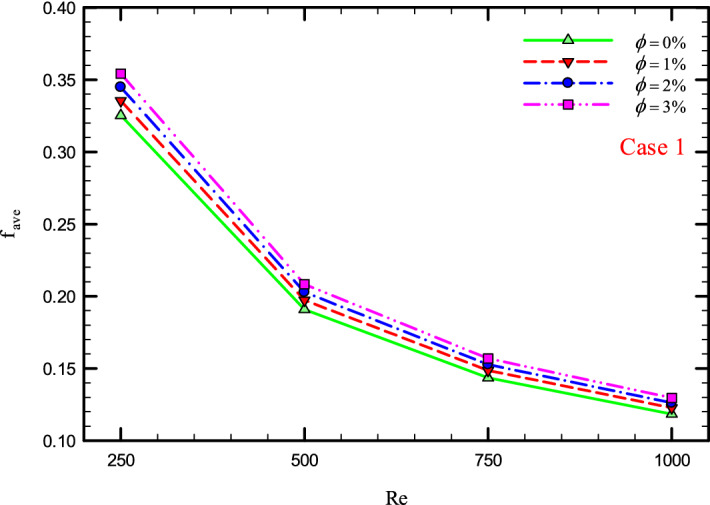
$f_{ave}$ versus Re for Case 1.

**Figure 38:**
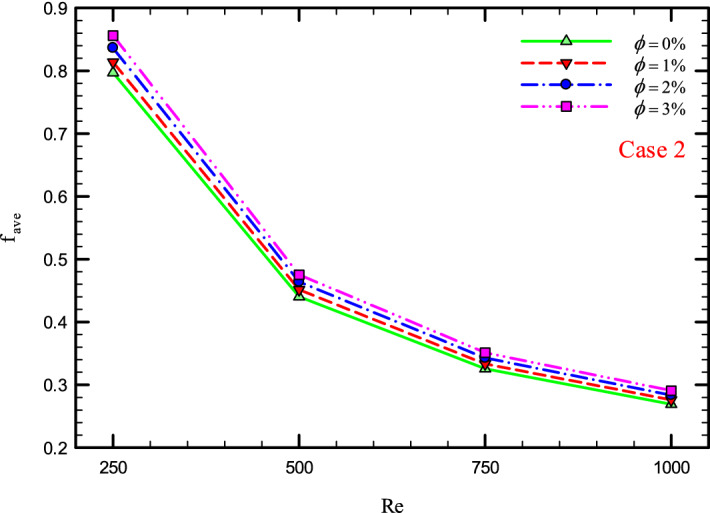
$f_{ave}$ versus Re for Case 2.

**Figure 39:**
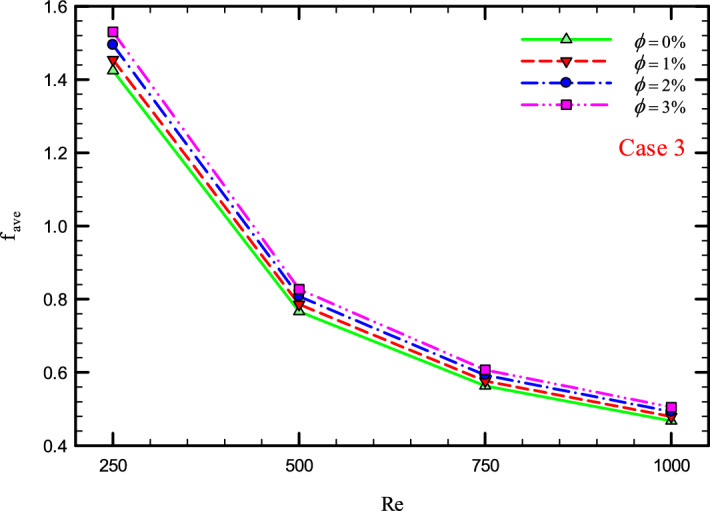
$f_{ave}$ versus Re for Case 3.

**Figure 40:**
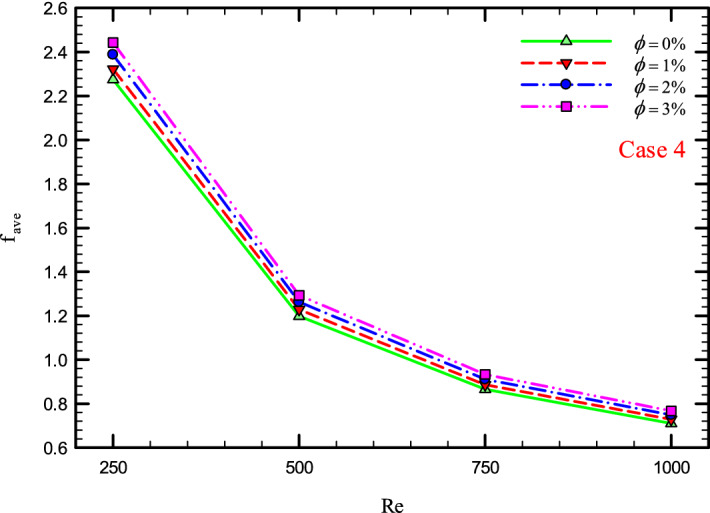
$f_{ave}$ versus Re for Case 4.

**Figure 41:**
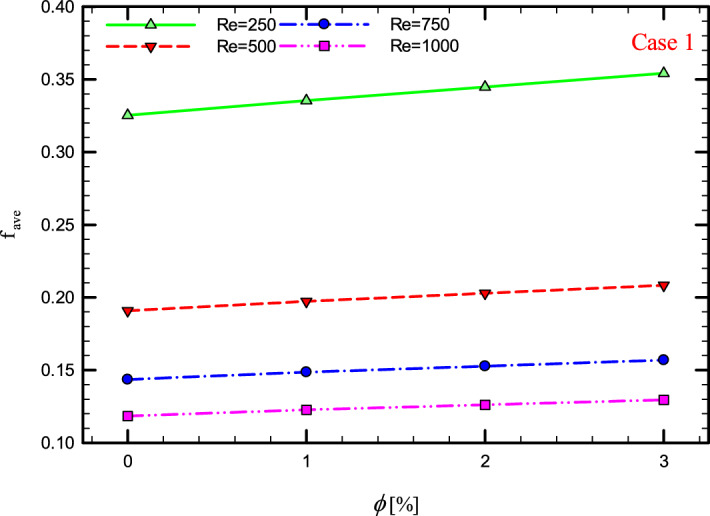
$f_{ave}$ versus $\phi$ for Case 1.

**Figure 42:**
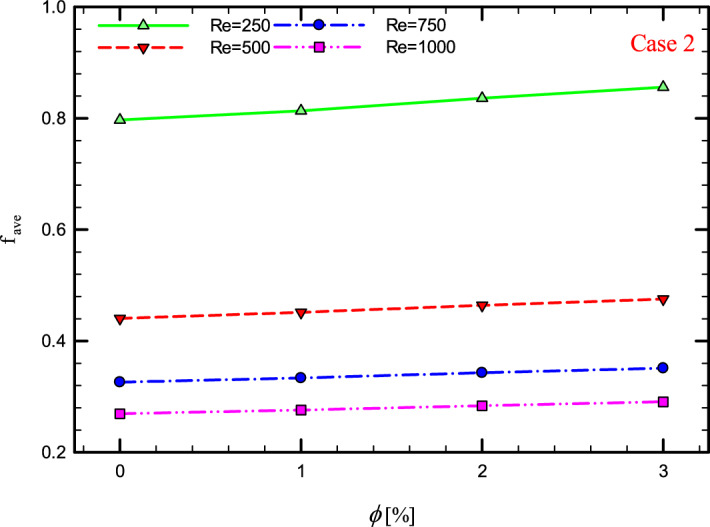
$f_{ave}$ versus $\phi$ for Case 2.

Figures 29, 30, 31, 32, 33, 34, 35, 36, 37, 38, 39, 40, 41 and 42 show the ${\text{Nu}}_{ave}$ versus geometry cases in Re = 250, 500, 750, and 1000, and $\phi =$ 0, 1, 2, and 3%. As can be seen, the highest value and the lowest value of the ${\text{Nu}}_{ave}$ in each $\phi$ and the *Re* are Case 4 and Case 1, respectively. It can be seen that the presence of the helical twisted tapes increases the ${\text{Nu}}_{ave}$, and this is exacerbated by the increase in the number of twisted tapes. Among the factors that can be mentioned for this increase in heat transfer is that: the presence of the twisted tapes causes the surface of the tube to shrink, and as a result, in a fixed *Re*, the fluid velocity increases to satisfy the conservation of mass.

### The effect of Re on $f_{x}^{{}}$

Figures 33, 34, 35 and 36 show a diagram of $f_{x}^{{}}$ for Re = 250, 500, 750, and 1000, $\phi =$ 0, 1, 2, and 3%, and the different shapes of the helical twisted tapes. The $f_{x}^{{}}$ in the pipe is inversely related to the *Re*, and as can be seen, the $f_{x}^{{}}$ decreases with increasing *Re*. The reason for this is the reduction in shear stress between the fluid and the walls due to the increase in the *Re*. As the *Re* increases, the velocity of the fluid at the inlet of the tube increases, which causes the inertial forces to overcome the viscous forces, thereby, reduces the $f_{x}^{{}}$.

### The effect of Re on $f_{ave}$

Figures 37, 38, 39 and 40 show the $f_{ave}$ versus *Re* and different $\phi$ for different cases.

### The effect of $\phi$ on $f_{ave}$

**Figure 43:**
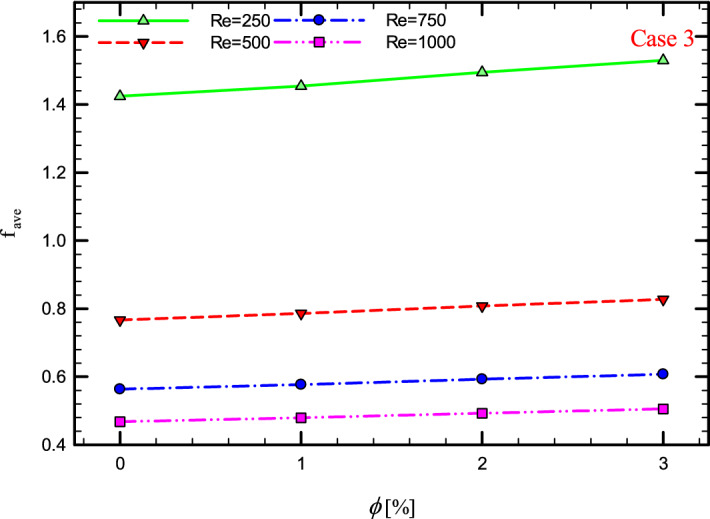
$f_{ave}$ versus $\phi$ for Case 3.

**Figure 44:**
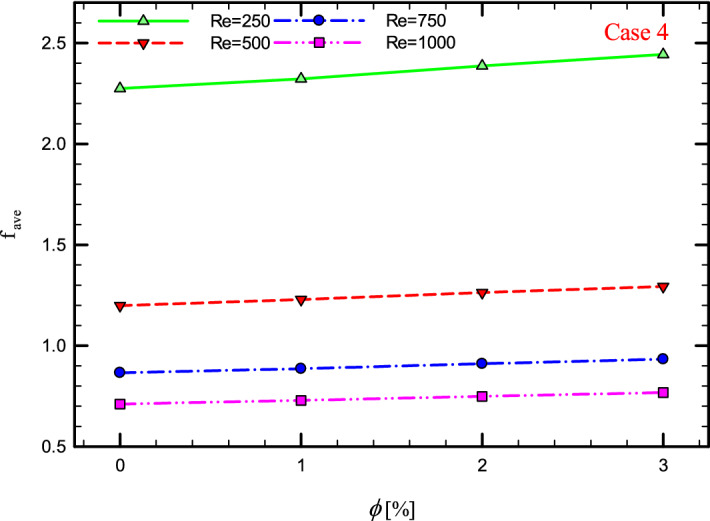
$f_{ave}$ versus $\phi$ for Case 4.

Figures 41, 42, 43 and 44 show the $f_{ave}$ versus $\phi$ in Re = 250, 500, 750, and 1000, and for different cases. As can be seen, increasing the $\phi$ causes the $f_{ave}$ to have an increasing trend in all geometric cases and all *Re*, and increasing the $\phi$ increases the $f_{ave}$. The reason for this increase can be attributed to the movement of nanoparticles in the base fluid, the increase in viscosity due to the presence of nanoparticles in the base fluid, and finally the change in velocity gradient near the wall.

### The effect of the number of helical twisted tapes on $f_{ave}$

**Figure 45:**
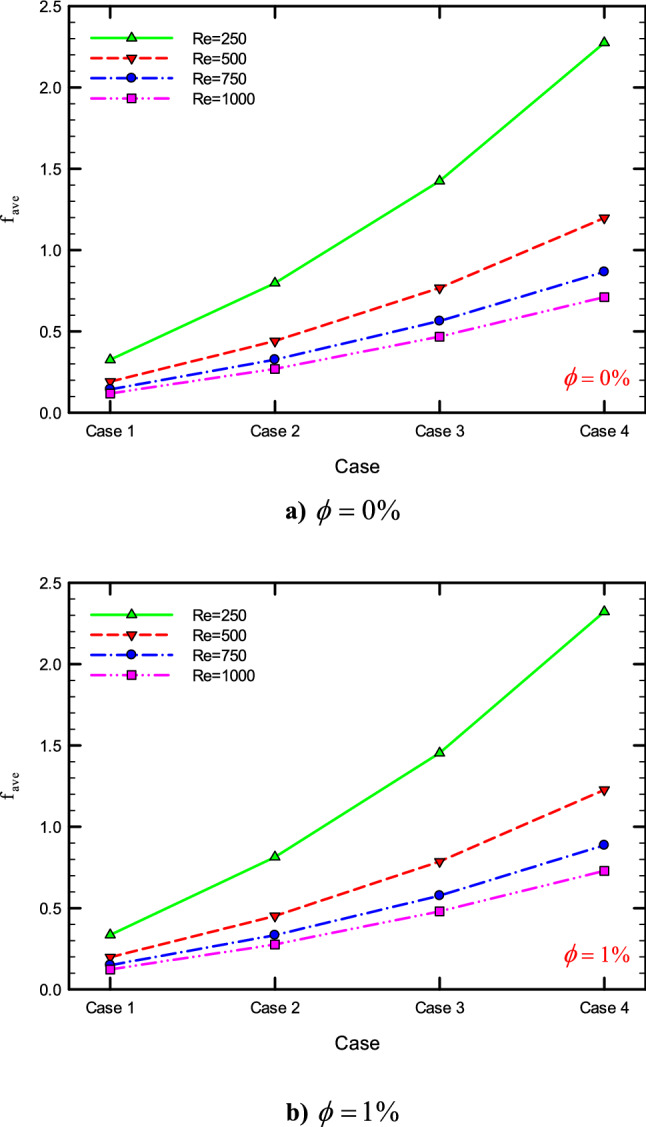

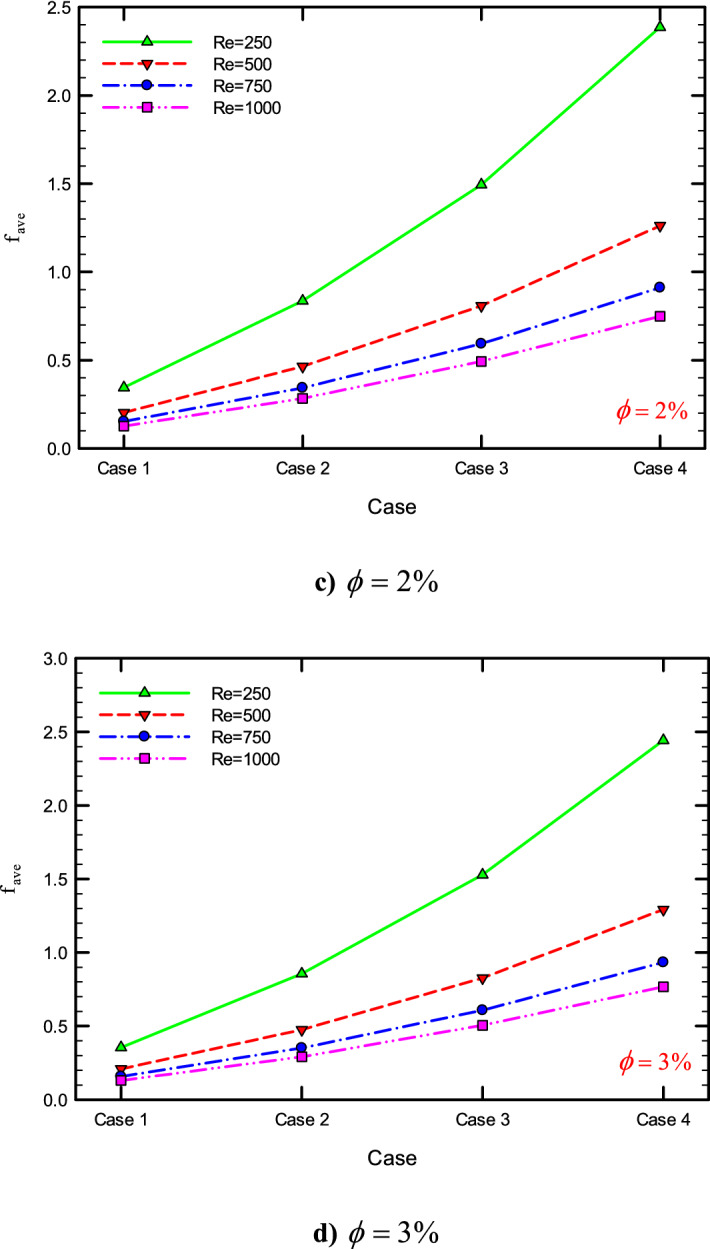
$f_{ave}$ for different cases i.

Figure 45 show the $f_{ave}$ versus the number of helical twisted tapes in different *Re* and $\phi$.

### Performance evaluation criterion (PEC)

**Figure 46:**
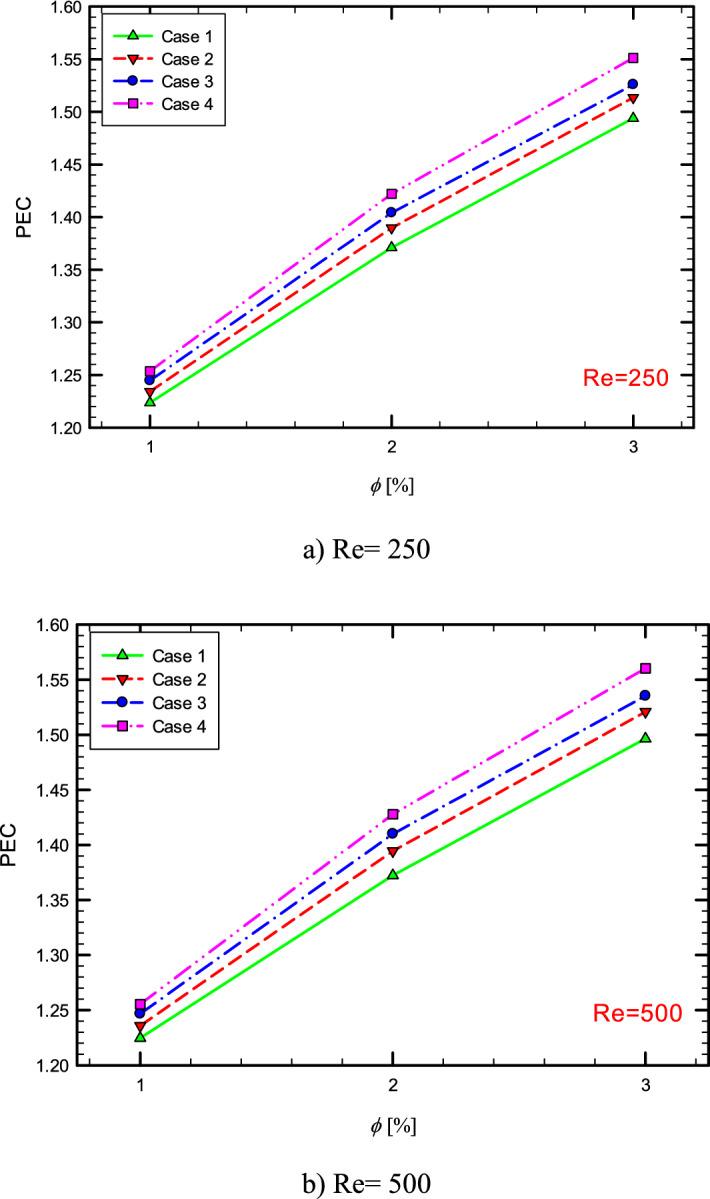

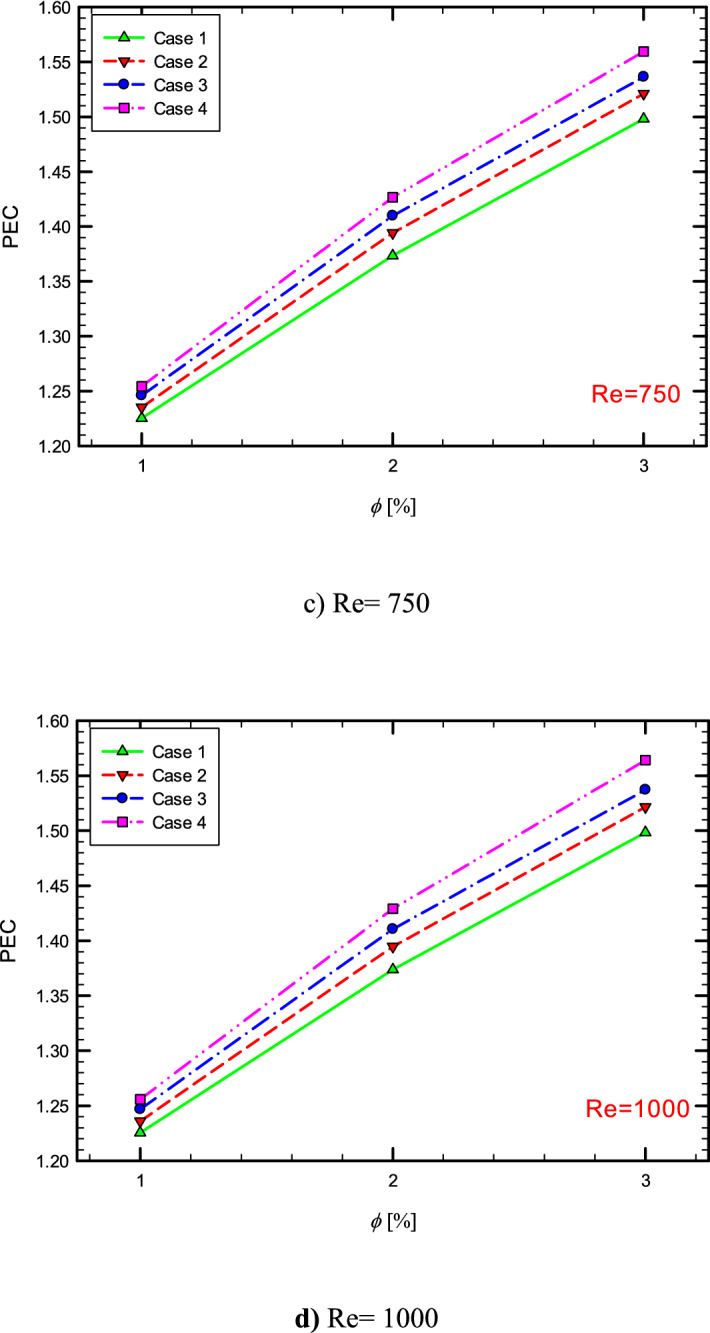
PEC versus $\phi$ at different Reynolds nimbers.

To examine whether the use of methods is appropriate from an engineering and economic point of view and whether it is appropriate to increase heat transfer to increase pressure drop, it is necessary to examine a quantity that is both hydrodynamic and thermally evaluate system performance. Therefore, in this section, the efficiency criterion is presented in different conditions. Performance Evaluation Criterion (PEC) is defined as follows^23–27^,

23 $$ PEC = \frac{{\left( {\frac{{Nu_{ave} }}{{Nu_{ave,s} }}} \right)}}{{\left( {\frac{f}{{f_{s} }}} \right)^{{\left( {1/3} \right)}} }} $$

where $Nu_{s}$ and $f_{s}$ are the Nusselt number and the friction factor in the pure fluid and $Nu_{ave}$ and f are the Nusselt number and friction factor in the tube in the different $\phi$^28,29^. If this coefficient is more than 1, the geometry has economic and engineering justification, and in cases where the PEC has the highest value, the most favorable conditions exist among the modes examined in the research. Figure 46 shows the PEC diagram versus $\phi$, respectively, in the flow inside the tube with one, two, and three twisted tapes. As can be seen, the presence of nanoparticles in the base fluid has increased the PEC.

## Conclusion

In this study, the nanofluid flow in a tube with helical twisted tapes with a two-phase model was investigated. The results obtained from numerical simulation indicate that:

- Increasing Re increases the $Nu_{ave}$ because as the Re increases, the velocity and fluid's momentum increase.
  - Increasing the Re reduces the friction factor because by increasing the Re, the inertial forces overcome the viscous forces and reduce the shear stress between the fluid and the walls.
  - Increasing the versus $\phi$ increases heat transfer because by increasing the $\phi$, the thermophysical properties of the base fluid increase.
  - Increasing the $\phi$ increases the $f_{ave}$, because they increase the viscous forces.
  - The presence of helical twisted tapes and an increase in their number increases the heat transfer due to the increase in the length of the flow path and the creation of severe secondary flows that cause the viscous sublayers to be disturbed.
  - The presence of helical twisted tapes increases the $f_{ave}$ because they increase the contact surface of the fluid and the walls.

## References

[1] Saysroy XA, Eiamsaard S (2017). Enhancing convective heat transfer in laminar and turbulent flow regions using multi-channel twisted tape inserts. Int. J. Therm. Sci..

[2] Saha S, Gaitonde U, Date A (1989). Heat transfer and pressure drop characteristics of laminar flow in a circular tube fitted with regularly spaced twisted-tape elements. Exp. Thermal Fluid Sci..

[3] Chun B-H, Kang HU, Kim SH (2008). Effect of alumina nanoparticles in the fluid on heat transfer in double-pipe heat exchanger system. Korean J. Chem. Eng..

[4] Date A, Saha S (1990). Numerical prediction of laminar flow and heat transfer characteristics in a tube fitted with regularly spaced twisted-tape elements. Int. J. Heat Fluid Flow.

[5] Wen D, Ding Y (2004). Experimental investigation into convective heat transfer of nanofluids at the entrance region under laminar flow conditions. Int. J. Heat Mass Transf..

[6] Sharma K, Sundar LS, Sarma P (2009). Estimation of heat transfer coefficient and friction factor in the transition flow with low volume concentration of Al2O3 nanofluid flowing in a circular tube and with twisted tape insert. Int. Commun. Heat Mass Transf..

[7] Murugesan P, Mayilsamy K, Suresh S (2010). Turbulent heat transfer and pressure drop in tube fitted with square-cut twisted tape. Chin. J. Chem. Eng..

[8] Jaisankar S, Radhakrishnan T, Sheeba K, Suresh S (2009). Experimental investigation of heat transfer and friction factor characteristics of thermosyphon solar water heater system fitted with spacer at the trailing edge of Left-Right twisted tapes. Energy Convers. Manage..

[9] Salman SD, Kadhum AAH, Takriff MS, Mohamad AB (2013). CFD analysis of heat transfer and friction factor characteristics in a circular tube fitted with quadrant-cut twisted tape inserts. Math. Probl. Eng..

[10] Salman SD, Kadhum AAH, Takriff MS, Mohamad AB (2014). Heat transfer enhancement of laminar nanofluids flow in a circular tube fitted with parabolic-cut twisted tape inserts. Sci. World J..

[11] Sun B, Zhang Z, Yang D (2016). Improved heat transfer and flow resistance achieved with drag reducing Cu nanofluids in the horizontal tube and built-in twisted belt tubes. Int. J. Heat Mass Transf..

[12] Hong Y, Du J, Wang S (2017). Turbulent thermal, fluid flow and thermodynamic characteristics in a plain tube fitted with overlapped multiple twisted tapes. Int. J. Heat Mass Transf..

[13] Hong Y, Du J, Wang S (2017). Experimental heat transfer and flow characteristics in a spiral grooved tube with overlapped large/small twin twisted tapes. Int. J. Heat Mass Transf..

[14] Toghraie D, Mahmoudi M, Akbari OA (2019). The effect of using water/CuO nanofluid and L-shaped porous ribs on the performance evaluation criterion of microchannels. Therm. Anal. Calorim..

[15] Samadifar M, Toghraie D (2018). Numerical simulation of heat transfer enhancement in a plate-fin heat exchanger using a new type of vortex generators. Appl. Therm. Eng..

[16] Arasteh, H., Mashayekhi, R. *et al.* Heat transfer enhancement by using CuO/water nanofluid in corrugated tube equipped with twisted tape. *Int. Commun. Heat Mass Transfer* **137**(3), 1045–1058 (2019).

[17] Maddah H, Alizadeh M, Ghasemi N, Alwi SRW (2014). Experimental study of Al2O3/water nanofluid turbulent heat transfer enhancement in the horizontal double pipes fitted with modified twisted tapes. Int. J. Heat Mass Transf..

[18] Eiamsa-ard S, Wongcharee K (2018). Convective heat transfer enhancement using Ag-water nanofluid in a micro-fin tube combined with non-uniform twisted tape. Int. J. Mech. Sci..

[19] Jafaryar M, Sheikholeslami M, Li Z (2018). CuO-water nanofluid flow and heat transfer in a heat exchanger tube with twisted tape turbulator. Powder Technol..

[20] Esfe MH, Mazaheri H, Mirzaei SS, Kashi E, Kazemi M, Afrand M (2018). Effects of twisted tapes on thermal performance of tri-lobed tube: An applicable numerical study. Appl. Therm. Eng..

[21] Sui Y, Teo C, Lee PS, Chew Y, Shu C (2010). Fluid flow and heat transfer in wavy microchannels. Int. J. Heat Mass Transf..

[22] Guo J, Fan A, Zhang X, Liu W (2011). A numerical study on heat transfer and friction factor characteristics of laminar flow in a circular tube fitted with center-cleared twisted tape. Int. J. Therm. Sci..

[23] Baragh S, Shokouhmand H, Ajarostaghi SS, Nikian M (2018). An experimental investigation on forced convection heat transfer of single-phase flow in a channel with different arrangements of porous media. Int. J. Therm. Sci..

[24] Baragh S, Shokouhmand H, Ajarostaghi SS (2019). Experiments on mist flow and heat transfer in a tube fitted with porous media. Int. J. Therm. Sci..

[25] Arefi-Oskoui, S., Khataee, A., Safarpour, M., Orooji, Y. & Vatanpour, V. A review on the applications of ultrasonic technology in membrane bioreactors. *Ultrason Sonochem* **58**, 104633 (2019). 10.1016/j.ultsonch.2019.104633 31450367

[26] Orooji, Y. *et al.* Preparation of mullite-TiB2-CNTs hybrid composite through spark plasma sintering. *Ceram. Int.* **45**, 16288–16296 (2019).

[27] Orooji, Y., *et al.* Facile fabrication of silver iodide/graphitic carbon nitride nanocomposites by notable photo-catalytic performance through sunlight and antimicrobial activity. *J. Hazard. Mater.* **389**, 122079 (2020). 10.1016/j.jhazmat.2020.122079 32062394

[28] NOrooji, Y., *et al.* Effects of ZrB2 reinforcement on microstructure and mechanical properties of a spark plasma sintered mullite-CNT composite. *Ceram. Int.* **45**, 16015–16021 (2019).

[29] Karimi-Maleh, H., Orooji, Y., Ayati, A. *et al.* Recent advances in removal techniques of Cr(VI) toxic ion from aqueous solution: A comprehensive review. *J. Mol. Liq.* **392**, 115062 (2021).

